# Composite adverse outcomes in obstetric studies: a systematic review

**DOI:** 10.1186/s12884-021-03588-w

**Published:** 2021-02-05

**Authors:** Dylan Herman, Kar Yee Lor, Abdul Qadree, Daphne Horn, Rohan D’Souza

**Affiliations:** 1 Institute of Medical Science, University of Toronto, Toronto, Ontario Canada; 2 Division of Maternal and Fetal Medicine, Department of Obstetrics and Gynaecology, Mount Sinai Hospital, University of Toronto, 600 University Avenue, Room 3-908, Toronto, Ontario M5G 1X5 Canada; 3 Lunenfeld-Tanebaum Research Institute, Mount Sinai Hospital, Toronto, Ontario Canada; 4 Faculty of Medicine, University of Aberdeen, Aberdeen, Scotland UK; 5 Department of Chemical and Physical Sciences, University of Toronto, Toronto, Ontario Canada; 6 Centre for Addiction and Mental Health (CAMH), Toronto, Ontario Canada

**Keywords:** Adverse pregnancy outcomes, Composite endpoints, Composite outcomes, Obstetric trials, Systematic review

## Abstract

**Background:**

Composite outcomes are increasingly being used in obstetric trials. The aim of this systematic review is to critically appraise the use of composite outcomes in obstetric RCTs with an intention of identifying limitations and providing potential solutions for future research.

**Methods:**

The study protocol was prospectively registered. Medline, Embase, Cochrane Databases and www.clinicaltrials.gov were searched for randomized controlled trials (RCTs) published in English between 1999 and 2019, using search terms related to pregnancy and composite outcomes. Study eligibility criteria: RCTs involving an obstetric condition that reported on a composite outcome. Study appraisal and synthesis methods: Screening and data extraction were performed in duplicate, and a descriptive synthesis and critical appraisal of composite obstetric outcomes, is presented.

**Results:**

Of the 4170 results screened, we identified 156 RCTs, reporting on 181 composite outcomes. Of these, 158 composite outcomes related to general morbidity and mortality, either exclusively maternal (*n*=20), fetal-neonatal [perinatal] (*n*=116) or maternal and perinatal (*n*=22) were included in the final analysis. Obstetric composite outcomes included between two and 16 components. Components that comprised these composite outcomes were often dissimilar in terms of severity and frequency of occurrence, unlikely to have similar relative risk reductions and sometimes unrelated to the study’s primary objective – important pre-requisites to consider while constructing composite outcomes. In addition, composite adverse obstetric outcomes often do not incorporate the perspectives of pregnant persons, embrace a holistic view of health or consider outcomes related to both members of the mother-fetus dyad.

**Conclusions:**

Composite outcomes are being increasingly used as primary outcomes in obstetric RCTs, based on which study conclusions are drawn and clinical recommendations made. However, there is a lack of consistency with regard to what components should be included within a composite adverse obstetric outcome and how these components should be measured. The use of novel research methods such as concept mapping may be able to address some of the limitations with the development of composite adverse obstetric outcomes, to inform future research.

**Supplementary Information:**

The online version contains supplementary material available at 10.1186/s12884-021-03588-w.

## Background

In randomized controlled trials (RCTs), the primary outcome is of distinct importance, as it directly relates to the primary objective of the study and indicates the efficacy of the treatment in question [1, 2]. In areas of medicine such as obstetrics, where serious clinical outcomes such as mortality and severe morbidity are fortunately very rare [1], composite endpoints such as a “composite adverse pregnancy outcome” are widely utilized in RCTs comparing the effectiveness of treatments. A composite outcome is one in which interrelated outcomes are combined into a single endpoint, in order to maximize the power to identify statistically significant predictors [2], such that participants experiencing any of the component outcomes are considered to have experienced the composite outcome [3]. Although composite outcomes are being widely used in obstetric RCTs, there is limited information on how the components of these composite outcomes are selected.

The aim of this study was to critically appraise the use of composite outcomes in obstetric RCTs with an intention of identifying limitations and providing potential solutions and recommendations for future research.

## Methods

The protocol for this review was prospectively registered on PROSPERO [4], the international prospective register of systematic reviews (CRD42019134852**)** and was conducted and reported in accordance with the Preferred Reporting Items for Systematic Reviews and Meta-Analyses (PRISMA) guidelines [5].

### Data sources

A medical information specialist with expertise in the conduct of systematic reviews of obstetric studies, prepared and ran the search on four bibliographic databases - MEDLINE, Embase, CENTRAL and www.clinicaltrials.gov. These databases were searched from January 1999 to January 2019, to identify published and ongoing obstetric RCTs that utilized composite outcomes, using keywords and Medical Subject Headings terms related to pregnancy, obstetrics, composite/combined, outcome/endpoint, and randomized controlled trials. The search strategy is attached as Supplementary Data 1.

### Eligibility criteria

All RCTs addressing an obstetric condition were included. Quasi-randomized or non-randomized studies, systematic reviews and all other study types were excluded. Conference abstracts that do not explicitly describe component outcomes within a composite, were also excluded. Authors of eligible studies were not contacted, as the aim of the study is to describe outcomes as reported by researchers in published manuscripts.

### Data extraction

Two reviewers (D.H., K.Y.L.) independently screened all titles and abstracts, and full-texts, and discrepancies were resolved through mutual discussion or adjudication by a third reviewer (RD), when required. Data was extracted by one reviewer (D.H.), using a piloted data extraction from, and a second reviewer (A.Q.) did do so for 10% of included studies to ensure accuracy. Extracted data included publication details, the condition studied, details on the composite outcome, for example, whether the composite was a primary or secondary outcome, whether the composite comprised maternal outcomes, fetal/neonatal (henceforth referred to as ‘perinatal’) outcomes, or both, and definitions of component outcomes.

### Assessment of risk of bias

No risk-of-bias tool to assess the quality of outcome reporting in RCTs is currently available. For this reason, and since the intent of this study was not to comment on the methodologic conduct of the study, but instead, to identify all composite outcomes and their components, a risk-of-bias assessment of the study’s methodologic quality was not performed.

### Data synthesis

We separated composite outcomes into those with only maternal components, only perinatal components and those that included outcomes related to both mother and baby. We then determined how many components were included in each composite, and the nature of these components. Based on the nomenclature used by the study investigators, and on the component outcomes, we further divided maternal composites outcomes into those related to general maternal morbidity vs. organ/condition-specific morbidity. For perinatal composites, we distinguished between those that included only antenatal events (morbidity and/or mortality), only neonatal/postnatal events, a combination of antenatal and postnatal events and those that also included infant events. We noted definitions for each component in order to determine whether they were defined in a standard manner between studies, thereby allowing appropriate meta-analysis.

## Results

### Study selection

**Fig. 1:**
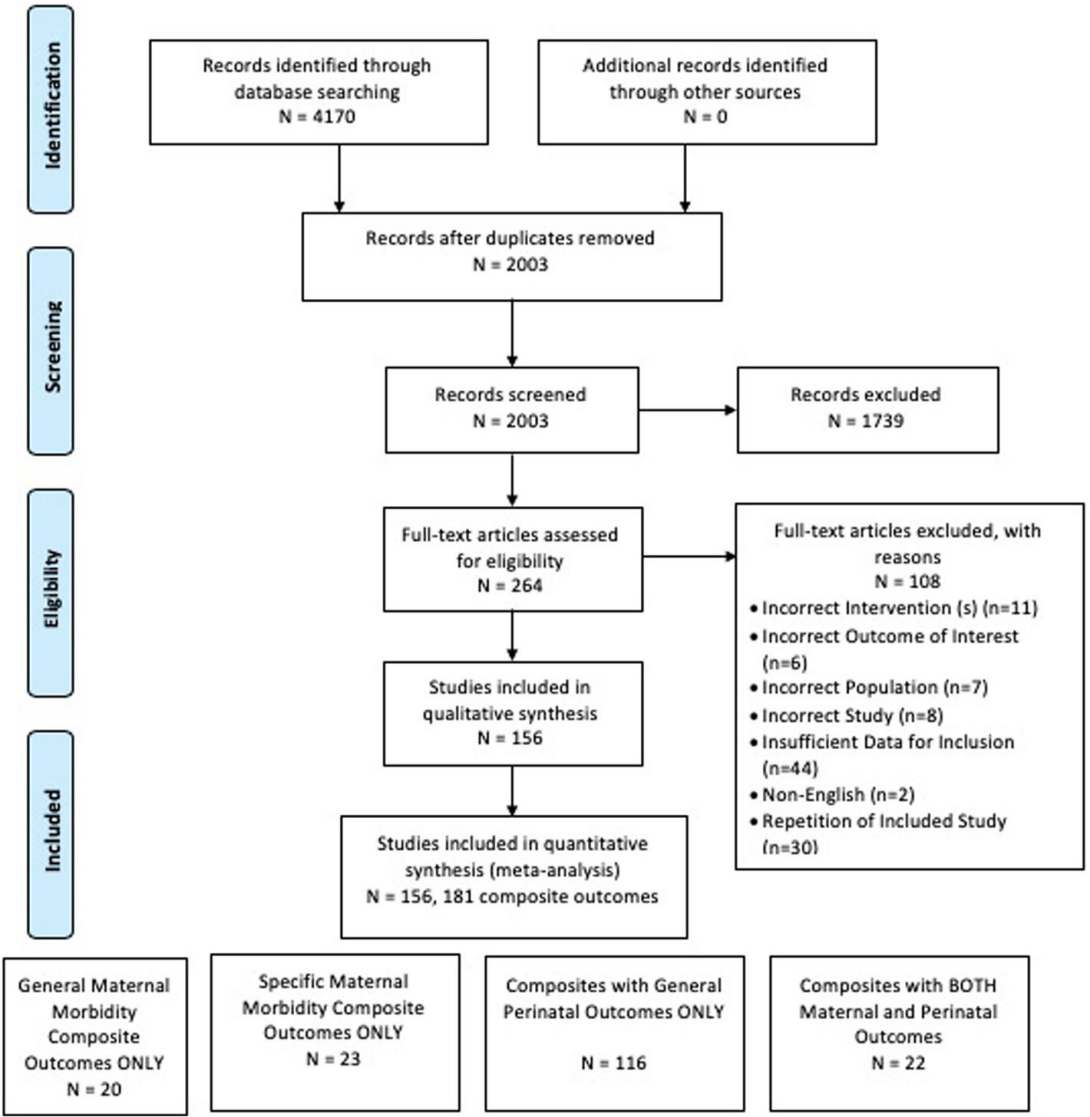
PRISMA diagram

The initial search yielded 4170 titles and abstracts, of which 2003 remained after excluding duplicates. Of these, 264 full-texts were reviewed in detail which resulted in 156 RCTs that reported 181 composite outcomes that were included in the final analysis (Fig. 1).

### Study characteristics

The characteristics of the 156 included RCTs are presented in Supplementary Data 2. Of these, 139 RCTs used a single composite outcome, while 13 RCTs used two, and four RCTs used three composite outcomes. The composite outcome was the primary outcome in 119 RCTs and the secondary outcome in 24 RCTs. Thirteen RCTs used composites both, as primary and secondary outcomes.

### Synthesis of results

The 156 included RCTs reported on 181 composite adverse outcomes, the details of which are described below.

#### Composite outcomes representing maternal health

Of the 181 composite outcomes described in obstetric RCTs, 43 only included maternal outcomes. These maternal composite outcomes belonged to two distinct categories – those describing general maternal morbidity and/or mortality (*n*=20), and those describing condition- or organ-specific maternal morbidity (*n*=23), and included between two and seven component outcomes.

##### Composite outcomes representing maternal morbidity and/or mortality

**Fig. 2:**
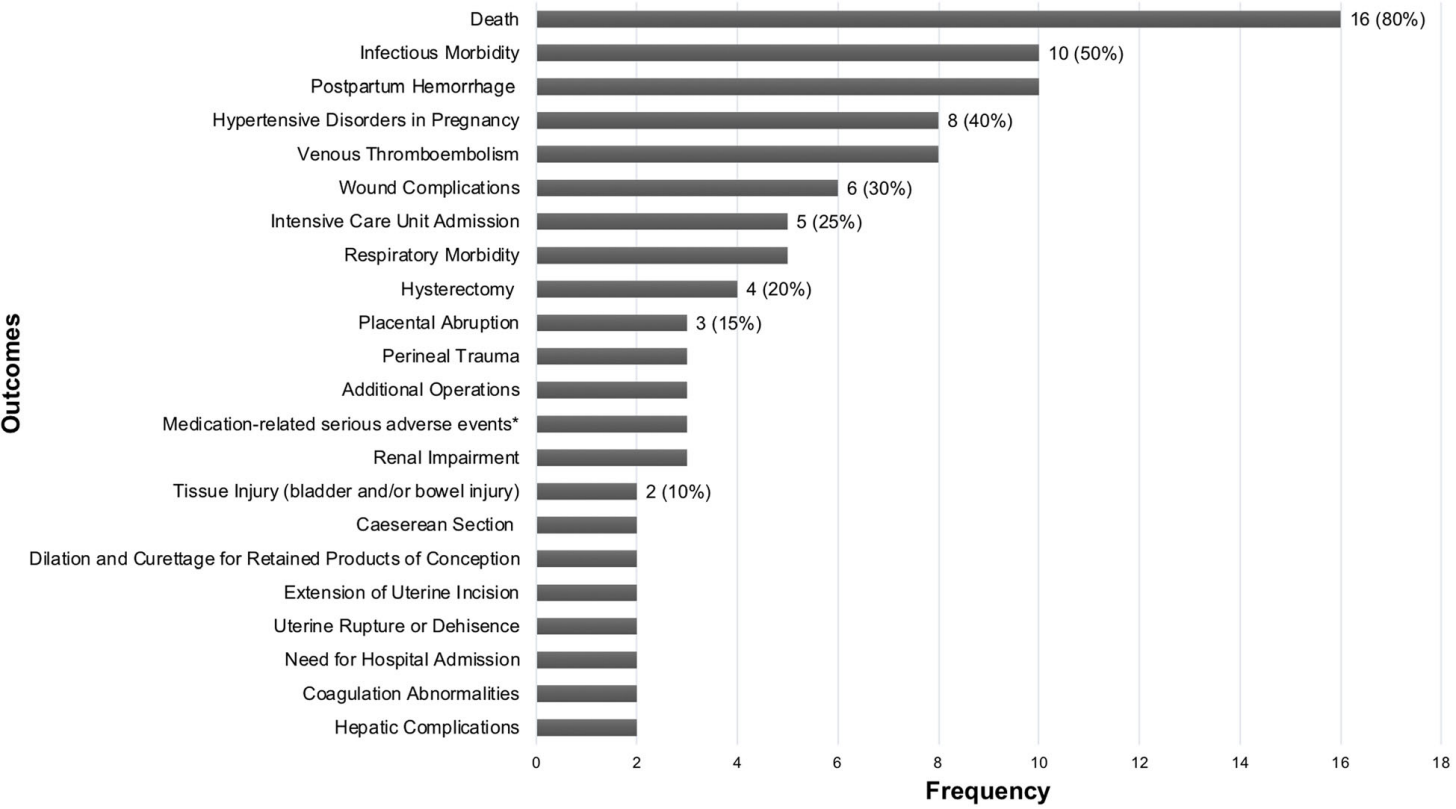
Components included in maternal morbidity/mortality composite outcomes. The following outcomes were included as part of only one composite outcome: cardiac complications, gestational diabetes mellitus, high dependency unit/postnatal stay, unscheduled visit to the emergency department or clinic, procedural or anaesthesia complication, cervical laceration, uterine inversion, operative vaginal delivery, identification or other source of symptoms, bowel obstruction, factors that might have complicated postpartum course. * includes serious allergic reaction and any serious event as a result of medication for e.g. cardiac events, pulmonary embolism and intensive care unit admission

**Fig. 3:**
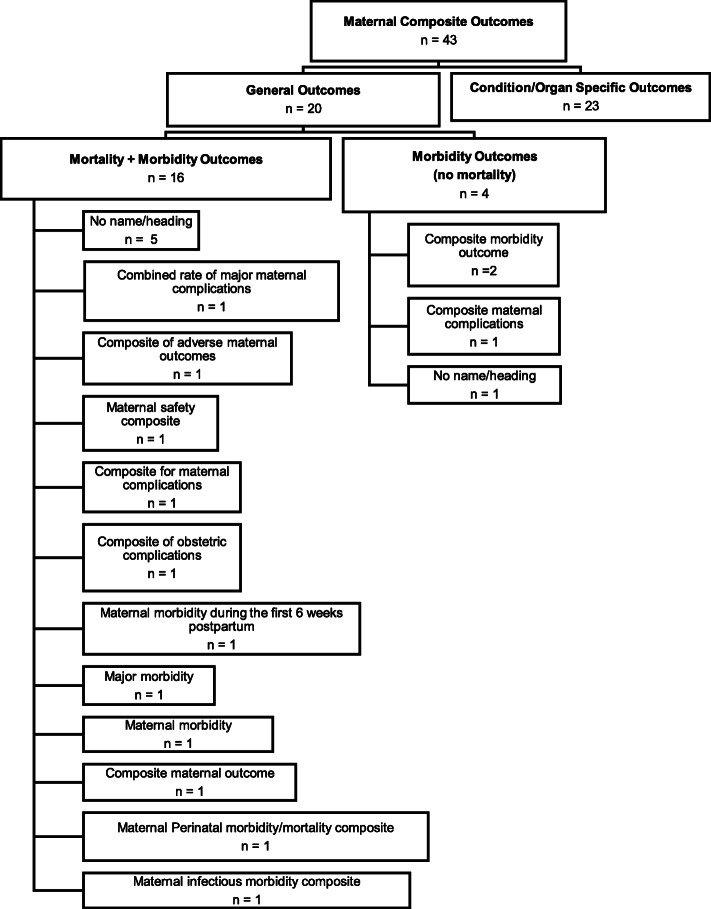
Maternal composite outcome labels

There were 20 composite outcomes in this subgroup, of which 16 included maternal mortality along with outcomes related to maternal morbidity. Of the four RCTs that used morbidity-related outcomes alone, one included mortality as an independent outcome (not part of the composite), but the other three did not consider mortality as one of the study’s outcomes [6–8]. The component outcomes are graphically represented in Fig. 2. Some important observations have been outlined below:

1. Nomenclature and component outcomes: 14/20 composite outcomes in this subgroup were labelled. Some of the common terms used to describe outcomes in this subgroup, included; ‘composite maternal complications’, ‘composite morbidity endpoint’ and ‘composite morbidity outcome’, although various combinations of descriptive terms related to severity (e.g. major, severe) and pregnancy (e.g. obstetric, pregnancy, maternal and perinatal) were used, as shown in Fig. 3. In some instances, the terminology used to describe the composite was not always representative of the component outcomes included. For example, ‘maternal infectious morbidity composite’, included outcomes that were not directly related to infectious morbidity such as postpartum hemorrhage requiring transfusion of greater than one unit up to six weeks and hospital readmission (for any reason) [9]. Another RCT, which included only outcomes related to infectious morbidity (fever, endometritis, sepsis, wound infection, wound complications and hospital readmission) labelled the outcome as ‘composite morbidity outcome’ [10], the same label used by another RCT to describe completely different component outcomes - postpartum hemorrhage (PPH), infection, unscheduled visits to the emergency department and need for hospital readmission [11]. Similarly, the term ‘major morbidity’ was used in two studies, both of which included maternal mortality as part of the composite, in addition to major morbidity [12, 13]. In one of these RCTs, ‘major morbidity’ was meant to refer to maternal death, postpartum hemorrhage greater than 2000 mL, intensive care unit (ICU) admission, hysterectomy and uterine inversion [12], while in another, it included death, eclampsia and Hemolysis, Elevated Liver Enzymes, Low Platelet (HELLP) Syndrome, placental abruption and organ-specific complications of hypertension [13].
2. Definitions and measurements of component outcomes: Another source of the variation, was how the component outcomes were defined/measured and the nature of sub-components. One classic example was related to the outcome PPH, which was either undefined or inconsistently defined. Definitions for PPH were sometimes based on the amount of blood lost (500 mL, 1000 mL, 1500 mL or 2000 mL) or the treatment received. Similarly, there was no consistency in what constituted hypertensive disorders of pregnancy (HDP). At least six different combinations of sub-component outcomes were identified in this study, ranging from the inclusion of eclampsia alone; eclampsia and HELLP syndrome; eclampsia, HELLP syndrome and severe preeclampsia (variably defined); HELLP syndrome and organ-specific complications of hypertension; preeclamptic toxemia and hypertension; and preeclamptic toxemia, eclampsia and severe non-proteinuric hypertension occurring within 37 weeks of gestation. Another example involves the composite outcome of infectious morbidity. This outcome when used, had eight different combinations of sub-components, which included sepsis, chorioamnionitis, endometritis, pneumonia, high-grade fever (variably defined), peritonitis, any infection within 30 days of delivery and postoperative wound infections. Further, two RCTs on the same topic, conducted by the same group of investigators, used almost identical component outcomes within their composite (VTE, pulmonary oedema and hypertensive disorders in pregnancy) [14, 15]; however, under hypertensive disorders of pregnancy outcome, one RCT included the sub-components of eclampsia, HELLP and severe preeclampsia while the other excluded severe preeclampsia. This difference, although subtle, could considerably affect the numbers of outcomes, and the ability to meta-analyze results.
3. Vague component outcomes: Some RCTs used generic or vague outcomes, which were inadequately defined, such as ‘factors that might have complicated the postpartum course’ [7] ‘subsequent identification of other source of symptoms’ [11], ‘any cardiac event’ and ‘any serious event as a result of medication’ [16]. The lack of clear definitions to describe these outcomes, in terms of both nature and prioritization affects reproducibility and the interpretation of study results.

##### Composite outcomes representing condition- or organ-specific morbidity

In addition to outcomes related to maternal mortality and general morbidity, there were 11 composite outcomes related to condition- or organ-specific morbidity including, general infectious morbidity (*n*=6), haemorrhagic complications (*n*=2) and one each related to respiratory morbidity, labour and delivery outcomes and perineal trauma. Condition or organ-specific morbidity composites are presented in Supplementary Data 4. Additionally, there were 12 composite outcomes specifically related to maternal wound morbidity, which are presented in Supplementary Data 3.

### Perinatal composite outcomes

**Fig. 4:**
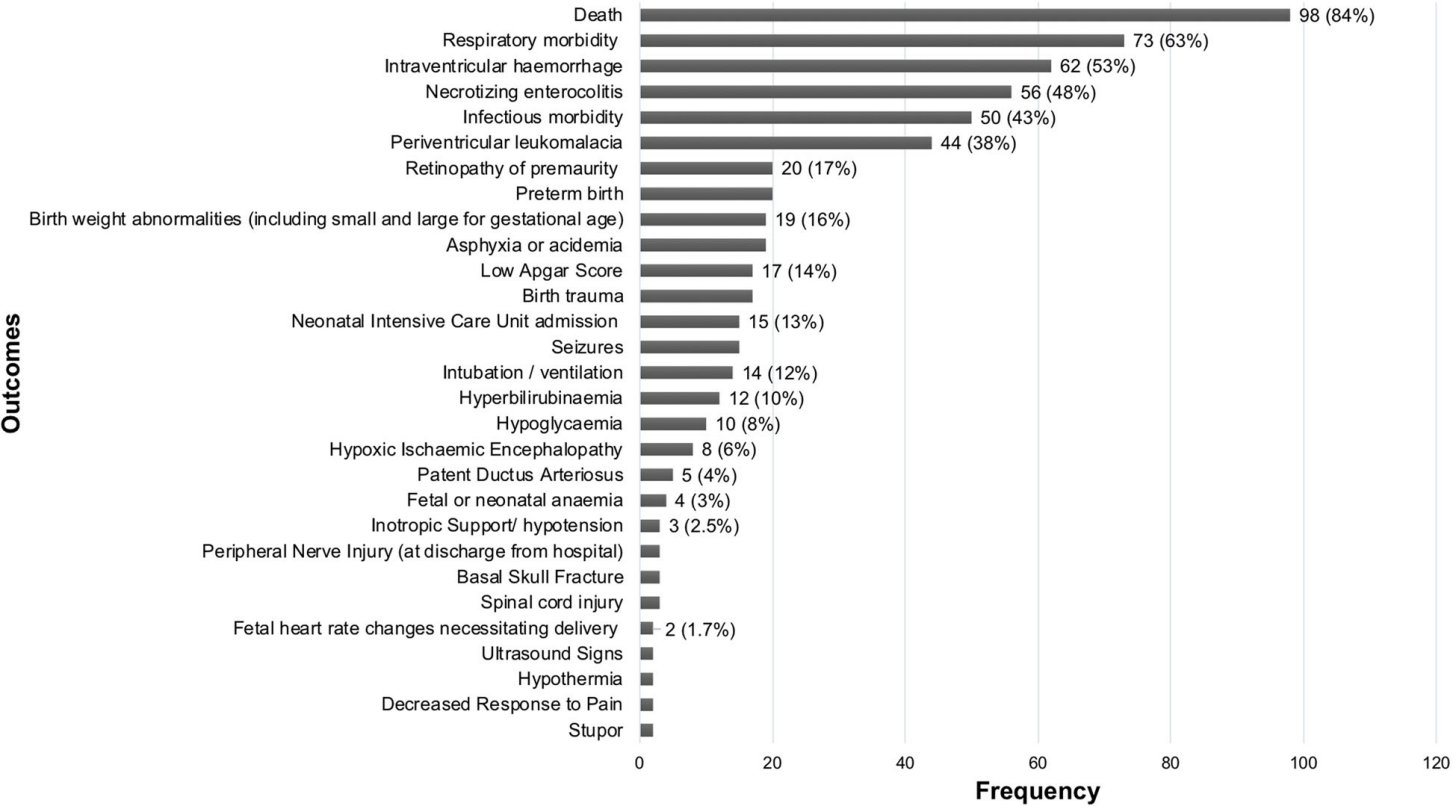
Components included in perinatal morbidity/mortality composite outcomes. The following outcomes were included as part of only one composite outcome: clinically significant genital injury, hypotonia, coma, tube feeding, loss to follow-up, congenital anomaly, ischemic injury, amniotic band syndrome, abnormal doppler findings on ultrasound, fluid abnormalities on ultrasound (oligohydramnios or polyhydramnios), TAPS, TTS reoccurrence, systemic inflammatory response syndrome, allergic reaction, transfer to long-term care facility, abnormal neurodevelopmental outcome at age 2 years, severe cerebral palsy at 2 years corrected age for prematurity

**Fig. 5:**
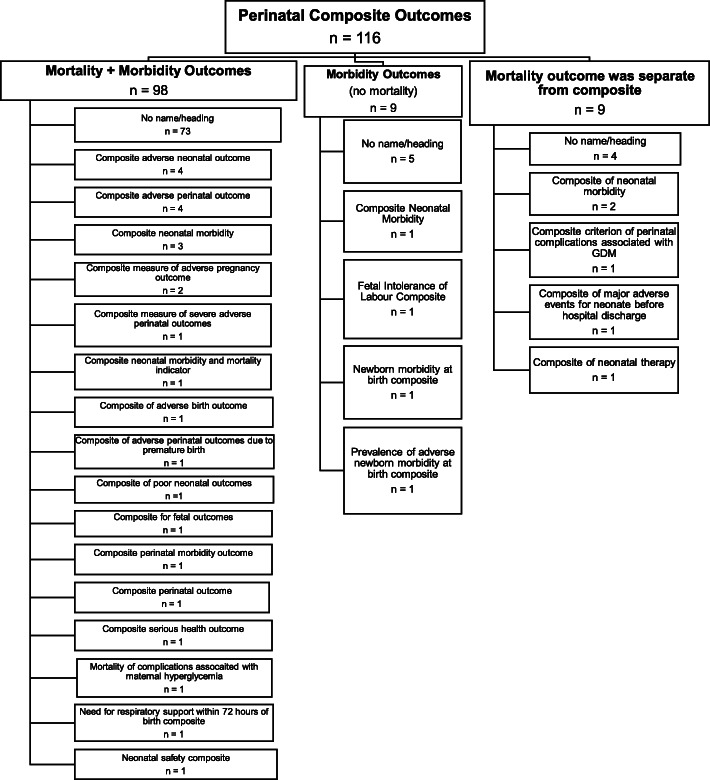
Perinatal composite outcome labels

The 116 outcomes representing fetal-neonatal (perinatal) adverse events, included between 2 and 14 components, the most frequent components being death (*n*=98), respiratory morbidity (*n*=73) and intraventricular hemorrhage (*n*=62). The distribution of component outcomes is graphically displayed in Fig. 4. Of the 116 composite outcomes, 98 included both mortality and morbidity, nine of which included morbidity outcomes and nine included mortality in an outcome separate from the composite. Variation in terminology to describe perinatal adverse composite outcomes is demonstrated in Fig. 5.

### Combined maternal-perinatal composite outcomes

**Fig. 6:**
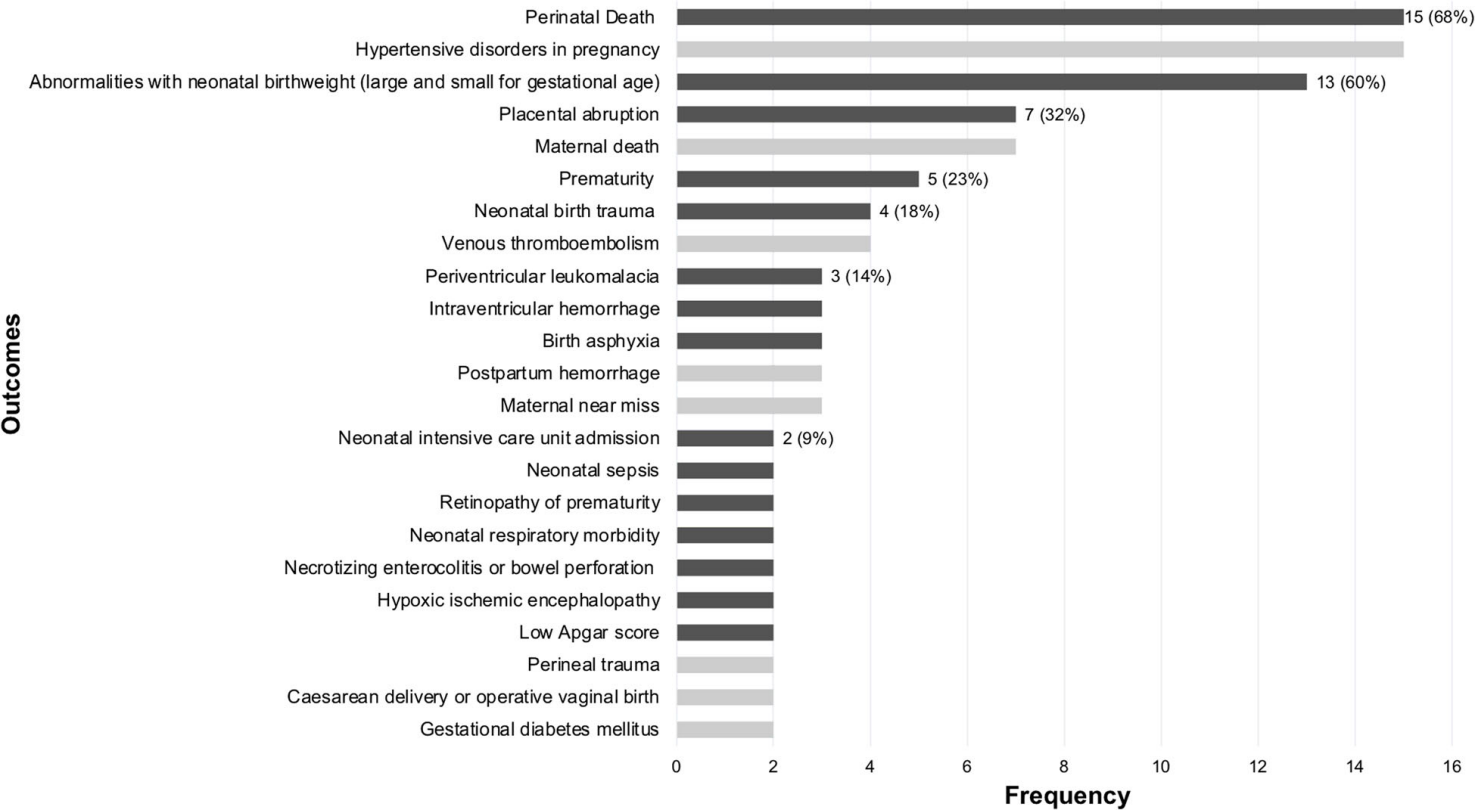
Components included in combined maternal-perinatal morbidity/mortality composite outcomes. The following outcomes were included as part of only one composite outcome: pulmonary edema, coagulopathy, maternal infection, abnormal maternal biomarkers, malpresentation, patent ductus arteriosus, renal dysfunction, postnatal administration of medications, neonatal hypoglycemia, neonatal hyperbilirubinemia, maternal anemia

The 22 combined maternal-perinatal composite outcomes included between two and 16 component outcomes, the most frequent of which were perinatal death and hypertensive disorders in pregnancy, both of which were included 15 times. The combined maternal-perinatal composite outcomes are graphically displayed in Fig. 6. The three composite outcomes in this group that were labelled were called ‘composite endpoint of late pregnancy complications’, ‘adverse pregnancy outcome’ and ‘composite score for perinatal complications’.

**Fig. 7:**
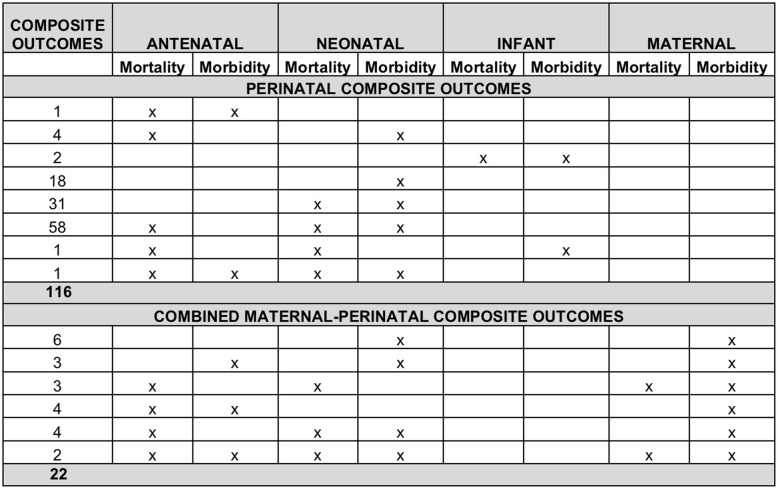
Distribution of maternal and perinatal endpoints within obstetric composite outcomes

Within each of the maternal, perinatal and combined groups of composite outcomes, the distribution of component outcomes related to mortality and morbidity for mother and baby are shown in Fig. 7. In addition to the inconsistencies identified under composite outcomes related to general morbidity and/or mortality above, there were a number of unique inconsistencies pertaining to composite outcomes involving the fetus and/or neonate. The perinatal period includes two distinct parts – antenatal (fetal period, prior to childbirth) and neonatal (following childbirth). Within these periods there are outcomes related to mortality (stillbirth or miscarriage in the antenatal period and early or late neonatal death in the neonatal period). There are also a distinct set of morbidity outcomes in the two periods, for example intrauterine growth restriction or fetal anaemia/hydrops in the antenatal period and respiratory morbidity or hypoxic ischaemic encephalopathy in the neonatal period. Of the 138 composites (116 perinatal and 22 combined) that included perinatal adverse outcomes i.e. antenatal and neonatal mortality and morbidity, only three RCTs (2.2%) reported composite outcomes related to antenatal and neonatal mortality and morbidity. The others included antenatal mortality (not morbidity) with neonatal mortality and morbidity (*n*=62); neonatal (but not antenatal) morbidity and mortality (*n*=31), only neonatal morbidity (*n*=25), and other combinations as depicted in Fig. 7. This figure also highlights the considerable variation in combined maternal-perinatal outcomes in terms of mortality and morbidity related to the mother, the fetus and the neonate.

Finally, it is also apparent that the composite outcomes used in obstetric trials do not adhere to principles that must be fulfilled by composite outcomes, which are as follows: 1) component outcomes within a composite endpoint should be clinically important and directly relevant to the primary objective of the trial [3, 17–22] and accurately portray the net effect of the treatment [3, 22], 2) component outcomes should be of similar importance [3, 18–20, 22], occur with similar frequency [22] and have similar relative risk reductions [17, 20, 22], 3) although components should be reported clearly and interpreted together [18, 21], individual outcomes must be reported in the secondary analysis [1, 3, 18, 21], 4) components should be prespecified in a published protocol/trial registration before commencement of the trials, and not changed once the trial commences [1–3, 17–19]. While many obstetric RCTs clearly pre-specify the component outcomes and adhere to principles of reporting the components together and separately as part of secondary analysis, the inclusion of rare and serious outcomes such as those related to mortality with those that occur frequently and non-life-threatening, and the pooling together of various outcomes that are sometimes unrelated to the primary objective of the study, means that obstetric trials do not always comply with all the principles surrounding the use of composite outcomes in clinical research.

## Discussion

This systematic review of 156 obstetric trials, which reported on 181 distinct composite outcomes highlights that although composite outcomes are being widely used in obstetric RCTs, there is a lack of consistency and comprehensiveness in the inclusion and definition of their component outcomes. This not only results in challenges with interpretation of study results, and comparing studies on the same topic, but also in determining the optimal interventions to inform clinical practice and policy. Although we acknowledge that every obstetric RCT is designed to answer a unique research question and not all RCTs can use an identical composite outcome, it is imperative that established principles are adhered to, while developing composite outcomes to ensure that the results of RCT truly reflect improvement in health as perceived by the health service user. The main limitations in relation to composite outcomes identified through this systematic review, and potential solutions are outlined below.

First, all outcomes considered within composite outcomes related to mortality and physiologic morbidity, which is reflective of the biomedical model of health. A systematic review undertaken to inform the World Health Organization intrapartum guidelines, identified that childbearing women value safety and psychosocial wellbeing equally (biopsychosocial model of health) and concluded that the design and provision of good quality maternity care should incorporate outcomes beyond just mortality and physiologic morbidity [23]. Improvement in levels of biomarkers, laboratory values, clinical measurements (such as blood pressure) and even some measures of morbidity (such as wound infection), may not translate to improvement in health as seen by pregnant persons, if accompanied by increase in adverse events, decline in functioning, long-term consequences or increased costs. There is therefore a move towards incorporating patient-reported outcomes such as quality of life as a primary outcome in other areas of women’s health [24]. A patient-reported outcome (PRO) is defined as “a measurement based on a report that comes directly from the patient (or study participant) about the status of particular aspects of or events related to a patient’s health condition” [25], the main characteristics of which include health-related quality of life, functional status, symptoms and symptom burden and experience of care [26]. Since pregnancy-specific instruments to measure these outcomes are now available [27], composite outcomes should consider components that represent a more holistic definition of health, as perceived by health service users, and not only researchers.

Second, obstetrics involves two distinct, yet intricately-related individuals – a mother and fetus, whose interests sometimes compete [28]. As identified in this systematic review, while reporting on morbidity and mortality, obstetric RCTs have traditionally considered maternal outcomes separately from perinatal outcomes, consigning outcomes related to the other member of the mother-fetus dyad as secondary outcomes. From the point of view of a pregnant person, some perinatal outcomes (for example a major congenital malformation or neonatal death) may be regarded as more important than some components of a primary composite of maternal morbidity (for example postpartum haemorrhage). Although many obstetric interventions affect both mother and baby, only 22 of the included composite outcomes in this systematic review considered both maternal and perinatal outcomes as part of a single composite adverse pregnancy outcome. Basing conclusions on outcomes related only to mother or baby, may not truly reflect a composite adverse obstetric outcome. Determining what outcomes pregnant persons consider important to include as part of a composite adverse pregnancy outcome, is important in this era of patient-centered care. While making clinical decisions, pregnant persons often need to make trade-offs which depend on the seriousness of consequences, as perceived by the pregnant person, and do not always agree with the perceptions of clinicians that often value maternal health over fetal health [28, 29]. Understanding how pregnant persons make these trade-offs, while considering mother and baby as one unit rather than two distinct entities, is vital to determining how outcomes are prioritized in a real-life setting, rather than packaging outcomes into those related to mother or baby or certain organ systems.

Both the above limitations can be addressed through the involvement of patients in determining how they prioritize outcomes. We propose doing this through the use of a novel research method called concept mapping, a participatory research method used to investigate wherein perspectives of participants are collected and integrated into a visual representation that demonstrates the relationship and differences between ideas [30, 31]. In the context of composite outcomes, by involving pregnant persons through various stages which include idea generation (focus groups), sorting and rating exercises and analysis, we will be able to determine how they prioritize various pregnancy outcomes, thereby providing researchers with a framework from which to choose relevant component outcomes while developing a composite outcome for a particular obstetric RCT.

Third, many of the composite outcomes used in obstetric RCTs did not fulfil the basic criteria for composite outcomes, which require the component outcomes should be clinically relevant, related directly to the primary objective of the trial, accurately portray the net effect of treatment, be of similar clinical severity, occur with similar frequency and have similar relative risk reductions [1–3, 17–22]. Failure to adhere to these principles can affect study conclusions, thereby influencing clinical practice and health policy. For example, some organizations have endorsed the administration of a single course of betamethasone for pregnant women between 34 0/7 weeks and 36 6/7 weeks of gestation at risk of preterm birth within 7 days, and who have not received a previous course of antenatal corticosteroids [32], based on a RCT which showed a significant reduction in a primary composite outcome of neonatal respiratory morbidity [33]. Other organizations recommended that antenatal corticosteroid therapy should not be administered to women at 36 0/7 to 36 6/7 who are at risk for preterm birth, and that even for those between 35 0/7 and 35 6/7, the balance of risks and benefits does not support routine administration of antenatal corticosteroid therapy [34]. One of the reasons for the disparate recommendations was that the component outcome ‘continuous positive airway pressure or high flow nasal cannula for ≥ 2 continuous hours’ which was largely responsible for the difference in the composite outcome between the two arms, was frequent but not felt to be as severe as the other components such as oxygen requirement with FiO2 of ≥30% for ≥4 continuous hours, mechanical ventilation, neonatal death, stillbirth or need for extracorporeal membrane oxygenation, none of which were significantly different between groups [34]. Although policy decisions are dependent on a number of factors, including resource utilization, costs, baseline populations and the relative prevalence of adverse outcomes in a certain settings, this example highlights how the inclusion of less severe, yet frequently occurring outcomes along with serious but rarer outcomes, within the same composite, can indirectly influence practice through the making of clinical recommendations. Finally, even when comparing the same interventions for the same condition, researchers tend to pick different component outcomes as part of the primary composite outcome, reflecting a lack of standardization in approach. For example, two RCTs studying the effectiveness of cervical pessary to prevent or reduce the rate of preterm birth, considered different components under ‘composite adverse perinatal outcome’ which consisted of: proven sepsis, intraventricular hemorrhage, necrotizing enterocolitis, retinopathy, bronchopulmonary dysplasia, respiratory distress syndrome, neonatal death [35] versus ‘perinatal death and severe perinatal morbidity including severe respiratory distress syndrome, bronchopulmonary dysplasia, necrotizing enterocolitis greater than stage I, grade III or IV intraventricular hemorrhage, periventricular leukomalacia greater than grade I, culture-proven sepsis and death before discharge from the hospital [36]. Variations between two studies in what component outcomes were included as ‘composite adverse perinatal outcome’ and how these components were defined could influence the interpretation of study results, and in turn can influence clinical practice and health policy. Projects addressing the limitations of standardization of outcome reporting and ensuring that composite outcomes fulfil essential pre-requisites are being undertaken under the auspices of the Outcome Reporting in Obstetric Studies (OROS) initiative [37].

This study had a number of strengths, which include a thorough overview of contemporary obstetric RCTs facilitated by the inclusion trials published over the past two decades as well as ongoing registered trials reflecting the current landscape, a critical review of composite outcomes from the methodologic standpoint and the identification of limitations and potential solutions to the development of composite adverse obstetric outcomes. The study’s limitations include the fact that risk-of-bias assessment of included RCTs was not undertaken. This was done intentionally, for a number of reasons. First, there is no risk-of-bias tool to assess the quality of outcome reporting. Second, all existing risk-of-bias instruments for clinical trials focus on study conduct and exclude outcome reporting and measurement. Third, our intent was to include all trials, to determine the scope of component outcomes included within adverse obstetric composite outcomes, regardless of study quality. The decision not to perform risk-of-bias assessment, therefore, does not affect study integrity.

## Conclusions

Although composite outcomes are being increasingly used in obstetric RCTs, composite adverse obstetric outcomes often do not incorporate the patient-perspective, embrace a holistic view of health, consider outcomes related to both members of the mother-fetus dyad, adhere to essential pre-requisites for developing composite outcomes or use standardized approaches to including and measuring component outcomes. Since this can influence study conclusions, clinical practice and health policy, measures to address these limitations are underway as part of the OROS initiative.

## Supplementary Information

**Additional file 1: Supplementary Data 1.** Search Strategy.

**Additional file 2: Supplementary Data 2.** Table of Included Studies (with references).

**Additional file 3: Supplementary Data 3.** Components outcomes included in maternal wound-related composite outcomes.

**Additional file 4: Supplementary Data 4.** Condition or organ-specific maternal composite outcomes.

## Data Availability

All data generated or analysed during this study are included in this published article (and its supplementary information files).

## Abbreviations

RCT: Randomized controlled trial
CAOOS: Composite Adverse Obstetric Outcomes Study
