# Supplementary material for: Composite adverse outcomes in obstetric studies: a systematic review
Source: BMC Pregnancy Childbirth. 2021 Feb 5;21:107. doi: 10.1186/s12884-021-03588-w (PMC7863533; doi:10.1186/s12884-021-03588-w)
Supplement: Supplementary file 1 — Additional file 1: Supplementary Data 1. Search Strategy. [file 12884_2021_3588_MOESM1_ESM.pdf]

Supplementary Data 1: Search Strategy

Medline Search Strategy

Exp Pregnancy/ or exp pregnancy complications/ or fetal mortality/ or infant mortality/ or maternal mortality/ or ((maternal or newborn or neonatal or perinatal) adj6 outcome*).ti,ab,kf. or pregnant women/ or exp fetus/ or exp infant, newborn/ or exp delivery, obstetric/ or peripartum period/ or postpartum period/ or exp pregnancy trimesters/ or breastfeeding/ or obstetrical surgical procedures/ or analgesia, epidural/ or exp infant, newborn, diseases/ or (pregnan* or postpartum or post-partum or peripartum or peri-partum or infant or newborn or neonatal or perinatal or peri-natal or postnatal or post-natal or antepartum or antenatal* or prenatal* or puerper* or amniocentes* or breastfe* or breast-fe* or lactation* or c?esarean or c?esarian or c?esarien or f?etal or f?etus).ti,ab,kf.

AND

(composite or factorial or combined specialty event or combined specialty events or combined effect or combined effects or combined end point or combined endpoint or combined endpoints or combined incidence or combined incidences or combined measure or combined occurrence or combined occurrences or combined outcome or combined outcomes or combined primary or combined rate or combined risk of or combined secondary).ti,ab,kf.

AND

(Randomized controlled trial or pragmatic clinical trial).pt. or exp randomized controlled trials as topic/ or “randomized controlled trial (topic)”/ or randomized controlled trial/ or randomization/ or random allocation/ or double-blind method/ or double blind procedure/ or double-blind studies/ or single-blind method/ or single blind procedure/ or single-blind studies/ or placebos/ or placebo/ or (random* or sham or placebo*).ti,ab,hw,kf. or ((singl* or double or tripl* or trebl*) adj (blind* or dumm* or mask*)).ti,ab,hw.kf.

NOT

Exp animals not humans/ or
(Calves or cows or sheep or bovine or ewes or pigs or piglets or mice or rats).ti,ab,kf.

Embase Search Strategy

1. ((tripl* or trebl*) adj (blind* or dumm* or mask*)).ti,ab,hw,kw.
2. ((singl* or doubl*) adj (blind* or dumm* or mask*)).ti,ab,hw,kw.
3. (random* or sham or placebo*).ti,ab,hw,kw.
4. Placebo/

5. Placebos/
6. Single-Blind Studies/
7. Single-Blind Method/
8. Single Blind Procedure/
9. Double-Blind Studies/
10. Double Blind Procedure/
11. Double-Blind Method/
12. Random Allocation/
13. Randomization/
14. Randomized Controlled Trial/
15. "Randomized Controlled Trial (topic)"/
16. exp Randomized Controlled Trials as Topic/
17. 1 or 2 or 3 or 4 or 5 or 6 or 7 or 8 or 9 or 10 or 11 or 12 or 13 or 14 or 15 or 16
18. (pregnan* or postpartum or post-partum or peripartum or peri-partum or infant* or newborn* or neonatal* or perinatal* or peri-natal or postnatal* or post-natal* or postpartum or antepartum or antenatal* or prenatal* or puerper* or amniocentes* or breastfe* or breast-fe* or lactation* or c?esarean or c?esarian or c?esarien or f?etal or f?etus).ti,ab,kw.
19. ((maternal or fetal or newborn or neonatal or perinatal) adj4 outcome*).ti,ab,kw.
20. exp pregnancy/
21. pregnancy disorder/
22. fetus mortality/ or infant mortality/ or maternal mortality/ or prenatal mortality/
23. exp pregnant woman/
24. exp fetus/
25. exp infant, newborn/
26. exp obstetric delivery/
27. perinatal period/
28. puerperium/
29. exp postnatal care/
30. perinatal care/
31. epidural analgesia/
32. newborn disease/
33. fetal therapy/
34. exp prenatal care/
35. fetus monitoring/
36. exp prenatal diagnosis/
37. labor pain/
38. 18 or 19 or 20 or 21 or 22 or 23 or 24 or 25 or 26 or 27 or 28 or 29 or 30 or 31 or 32 or 33 or 34 or 35 or 36 or 37
39. (composite or factorial or combined specialty event* or combined effect or combined effects or combined end point or combined end points or combined endpoint or combined endpoints or combined incidence* of or combined measure* or combined occurrence* or combined outcome* or combined primary

or combined rate or combined rates or combined risk of or combined risks of or combined secondary).ti,ab,kw.
40. exp animal/ not human/
41. (calves or cows or sheep or bovine or ewes or pigs or piglets or mice or rats).ti,ab,kw.
42. 17 and 38 and 39
43. 42 not 40
44. 43 not 41
45. limit 44 to english language
46. remove duplicates from 45

<u>Cochrane Central Search Strategy</u>
1. Pregnancy/
2. exp pregnancy complications/
3. fetal mortality/ or infant mortality/ or maternal mortality/ or perinatal mortality/
4. ((maternal or fetal or newborn or neonatal or perinatal) adj6 outcome*).ti,ab,kw.
5. Pregnant Women/
6. exp Fetus/
7. exp infant, newborn/
8. exp Delivery, Obstetric/
9. peripartum period/ or postpartum period/ or exp pregnancy trimesters/
10. Analgesia, Epidural/
11. exp Fetal Therapies/
12. exp Fetal Monitoring/
13. perinatal care/ or postnatal care/
14. Labor Pain/
15. (pregnan* or postpartum or post-partum or peri-partum or peripartum or infant or newborn or neonatal* or perinatal* or peri-natal* or postnatal* or post-natal* or postpartum or antepartum or anetnatal* or prenatal* or puerper* or amniocentes* or breastfe* or breast-fe* or lactation* or c?esarean or c?esarian or c?easarien or f?etus or f?etal).ti,ab,kw.
16. 1 or 2 or 3 or 4 or 5 or 6 or 7 or 8 or 9 or 10 or 11 or 12 or 13 or 14 or 15
17. (composite or factorial or combined specialty events or combined specialty event or combined effect or combined effects or combined end point or combined end points or combined endpoint or combined endpoints or combined incidence* or combined measure* or combined occurrence* or combined outcome* or combined primary or combined rate or combined rates or combined risk or combined risks or combined secondary).ti,ab,kw.
18. 16 and 17
19. exp animals/ not humans/
20. exp animals/ or dogs/
21. (calves or cows or cow or sheep or bovine or ewe or ewes or pigs or piglets or mice or rats).ti,ab,kw.
22. 18 not 21
23. 22 not 19
