# Supplementary material for: Composite adverse outcomes in obstetric studies: a systematic review
Source: BMC Pregnancy Childbirth. 2021 Feb 5;21:107. doi: 10.1186/s12884-021-03588-w (PMC7863533; doi:10.1186/s12884-021-03588-w)
Supplement: Supplementary file 2 — Additional file 2: Supplementary Data 2. Table of Included Studies (with references). [file 12884_2021_3588_MOESM2_ESM.pdf]

Supplementary Data 2: Table of Included Studies – (with references)

Year	Author (first author only)	Study Title / Topic / Condition	Type of Composite	Primary or Secondary Outcome?
2019	Pels[1]	sildenafil versus placebo for pregnant women with severe early onset fetal growth restriction (STRIDER)	Perinatal	Primary
2019	Pasquier[2]	Intentional early delivery versus expectant management for preterm premature rupture of membranes at 28-32 weeks' gestation (MICADO STUDY)	Perinatal	Primary
2018	Baqui[3]	Prevalence of and risk factors for abnormal vaginal flora and its association with adverse pregnancy outcomes in a rural district in north-east Bangladesh	1. Maternal - General<br>2. Perinatal	Primary
2018	Simon[4]	Supplemental oxygen room air during planned caesarean delivery on umbilical cord gases	1. Maternal - General<br>2. Perinatal	Primary
2018	Simmons[5]	Immediate treatment for GDM (intervention) or no treatment (control): Treatment of Booking Gestational diabetes Mellitus (TOBOGM)	Combined	Primary
2018	Venkateswaran[6]	eRegQual-an electronic health registry with interactive checklists and clinical decision support for improving quality of antenatal care: protocol	Combined	Primary

Supplementary Data 2: Table of Included Studies – (with references)

2018	Goldenberg[7]	Routine antenatal ultrasound in low- and middle-income countries	Combined	Primary
2018	Luitjes[8]	Innovative implementation strategy and feedback versus minimal implementation strategy of audit and feedback only.	Maternal - General	Primary
2018	Nathan[9]	Evaluation of a novel device for the management of high blood pressure and shock in pregnancy in low-resource settings: protocol (CRADLE-3 trial)	Maternal - General	Primary
2018	Zaki[10]	Comparison of staples vs subcuticular suture in class III obese women undergoing cesarean	Maternal - Wound	Primary
2018	Scrafford[11]	Effect of intra-operative glove changing during cesarean section on post-operative complications	Maternal - Wound	Primary
2018	Armstrong-Buisseret[12]	(ReMIT-2 protocol): standard care informed by placental growth factor (PlGF) blood test result versus standard care alone in women presenting with reduced fetal movement at or after 36+ 0 weeks gestation	Perinatal	Primary
2018	Berry[13]	insulin plus metformin versus insulin plus placebo for the treatment of type 2 diabetes complicating pregnancy (MOMPOD) study	Perinatal	Primary

Supplementary Data 2: Table of Included Studies – (with references)

2018	Norman[14]	no pessary vs (Arabin) pessary to prevent preterm birth in twin pregnancy with health economics and acceptability: STOPPIT-2-a protocol	Perinatal	Primary
2018	Chappell[15]	Ursodeoxycholic acid versus placebo in the treatment of women with intrahepatic cholestasis of pregnancy (ICP) to improve perinatal outcomes: Protocol (PITCHES)	Perinatal	Primary
2018	Subtil[16]	single-course or triple-course clindamycin for bacterial vaginosis in pregnancy (PREMEVA)	Perinatal	Primary
2018	Brocklehurst[17]	Computerised interpretation of the fetal heart rate during labour: Decision support or no decision support (INFANT)	Perinatal	Primary
2018	Hermans[18]	Cervical pessary vs no pessary after arrested preterm labor	Perinatal	Primary
2018	Cahill[19]	Immediate vs Delayed Pushing on Rates of Spontaneous Vaginal Delivery among Nulliparous Women Receiving Neuraxial Analgesia	Perinatal	Primary
2018	Dugoff[20]	Prevention of preterm birth with pessary vs no pessary in singletons (PoPPS):	Perinatal	Primary
2018	Blackwell[21]	PROLONG Clinical Hydroxyprogesterone Caproate to Reduce Recurrent Preterm Birth	Perinatal	Primary

Supplementary Data 2: Table of Included Studies – (with references)

2018	Grobman[22]	Labor induction versus expectant management in low-risk nulliparous women	Perinatal	Primary
2018	Senat[23]	Effect of Glyburide vs subcutaneous insulin on perinatal complications among women with gestational diabetes	Perinatal	Primary
2018	Balogun[24]	Serial Third-Trimester Ultrasonography Compared With Routine Care in Uncomplicated Pregnancies	1. Maternal - General<br>2. Maternal - General<br>3. Perinatal	Primary and Secondary
2018	Norman[25]	progesterone prophylaxis vs placebo to prevent preterm labour improve outcome? A randomised double-blind placebo-controlled trial (OPPTIMUM)	1. Perinatal<br>2. Perinatal	Primary and Secondary
2018	Price[26]	Health consequences for mother and baby of substantial vs modest pre-conception weight loss in obese women: protocol	Combined	secondary
2018	Wihbey[27]	Prophylactic negative pressure therapy compared with standard surgical dressings on Wound Complication after Cesarean Delivery in Women with Class II or III Obesity	Maternal - Wound	Secondary
2017	Bardou[28]	NAITRE- effect of conditional cash transfer on poor pregnancy outcomes in underprivileged women: Protocol	Combined	Primary

Supplementary Data 2: Table of Included Studies – (with references)

2017	Schreiber[29]	(HYPATIA) Protocol: Hydroxychloroquine versus Placebo in Addition to Standard Treatment in Pregnant Women with Antiphospholipid Syndrome or Antibodies	Combined	Primary
2017	Fransen[30]	Simulation-based team training for multi-professional obstetric care teams to improve patient outcome:	Combined	Primary
2017	Linden[31]	Pelvic Examination vs no pelvic examination in Patients Presenting to the Emergency Department With Vaginal Bleeding or Abdominal Pain When an Intrauterine Pregnancy Is Identified on Ultrasonography	Maternal - General	Primary
2017	Ruhstaller[32]	Prophylactic Wound Vacuum Therapy vs standard wound care after Cesarean Section to Prevent Wound Complications in the Obese Population: A (the ProVac Study)	Maternal - Wound	Primary
2017	Pasquier[33]	A cluster-randomized trial to reduce major perinatal morbidity among women with one prior cesarean delivery in Quebec (PRISMA trial): protocol	Perinatal	Primary
2017	van Zijl[34]	Pessary or Progesterone to Prevent Preterm delivery in women with short cervical length: The Quadruple P	Perinatal	Primary

Supplementary Data 2: Table of Included Studies – (with references)

2017	Berghella[35]	BiotequeTM cup cervical pessary or no pessary for prevention of preterm birth with pessary in twins (PoPPT)	Perinatal	Primary
2017	Dodd[36]	Fetal middle cerebral artery Doppler to time second and subsequent intrauterine transfusions to treat anaemia due to red cell allo-immunisation	Perinatal	Primary
2017	Semrau[37]	Outcomes of a coaching-based WHO safe childbirth checklist program in India	Combined	Primary and Secondary
2017	Groom[38]	standard high-risk care or standard high-risk care plus enoxaparin for the prevention of preeclampsia and intrauterine growth restriction in women with a history	Combined	Primary and Secondary
2017	Springel[39]	chlorhexidine-alcohol versus povidone-iodine for cesarean antisepsis	Maternal - Wound	Secondary
2017	Koullali[40]	cervical pessary or cervical cerclage in the prevention of preterm delivery in women with short cervical length and a history of preterm birth - PC study	Perinatal	Secondary
2017	Rodo[41]	Arabin cervical pessary vs expectant management for prevention of preterm birth in cases of twin-to-twin transfusion syndrome treated by fetoscopic LASER coagulation: The PECEP LASER	Perinatal	Secondary

Supplementary Data 2: Table of Included Studies – (with references)

2017	Visser[42]	Low dose aspirin vs placebo in the prevention of recurrent spontaneous preterm labour - the APRIL study	Perinatal	Secondary
2017	Crowther[43]	Vaginal progesterone pessaries for pregnant women with a previous preterm birth to prevent neonatal respiratory distress syndrome (the PROGRESS Study)	Perinatal	Secondary
2017	Connolly[44]	simultaneous (oxytocin and Foley balloon) or sequential (oxytocin after the expulsion of Foley balloon) induction of Labor Trial in Multiparas (FIAT-M)	Perinatal	Secondary
2016	Wattar[45]	Effect of simple, targeted diet in pregnant women with metabolic risk factors on maternal and fetal outcomes (ESTEEM): protocol	1. Maternal - General<br>2. Perinatal	Primary
2016	Barasinski[46]	Effect of the type of maternal pushing during the second stage of labour on obstetric and neonatal outcome: The EOLE protocol	1. Maternal - General<br>2. Maternal - General	Primary
2016	Salim[47]	Adjusting enoxaparin dosage according to anti-fxa levels vs 40 mg daily enoxaparin nonpregnancy outcome in thrombophilic women	Combined	Primary
2016	Feig[48]	Metformin or placebo in women with type 2 diabetes in pregnancy (MiTy)	Combined	Primary

Supplementary Data 2: Table of Included Studies – (with references)

2016	Kimani[49]	azithromycin-chloroquine versus sulfadoxine-pyrimethamine for intermittent preventive treatment of plasmodium falciparum malaria infection in pregnant women in Africa	Combined	Primary
2016	Haddad[50]	Enoxaparin and Aspirin Compared with Aspirin Alone to Prevent Placenta-Mediated Pregnancy Complications	Combined	Primary
2016	Madanitsa[51]	Scheduled Intermittent Screening with Rapid Diagnostic Tests and Treatment with Dihydroartemisinin-Piperaquine versus Intermittent Preventive Therapy with Sulfadoxine-Pyrimethamine for Malaria in Pregnancy in Malawi	Combined	Primary
2016	McCarthy[52]	Self-weighing and simple dietary advice for overweight and obese pregnant women to reduce obstetric complications without impact on quality of life	Maternal - General	Primary
2016	Ducloy-Bouthors[53]	Fibrinogen concentrate vs placebo for postpartum haemorrhage-induced coagulopathy: protocol (FIDEL)	Maternal - Wound	Primary
2016	Duggal[54]	Perioperative Oxygen Supplementation and Surgical Site Infection After Cesarean Delivery	Maternal - Wound	Primary
2016	Van Vliet[55]	Nifedipine versus atosiban for threatened preterm birth (APOSTEL III)	Perinatal	Primary

Supplementary Data 2: Table of Included Studies – (with references)

2016	Norman[56]	Vaginal progesterone prophylaxis for preterm birth (the OPPTIMUM study)	Perinatal	Primary
2016	Eikelder[57]	Induction of labour at term with oral misoprostol versus a Foley catheter (PROBAAT-II):	Combined	Primary
2016	Murphy[58]	The Breathing for Life Trial: A of fractional exhaled nitric oxide (FENO)-based management of asthma vs clinical assessment and self-management education during pregnancy and its impact on perinatal outcomes and infant and childhood respiratory health	Perinatal	Primary
2016	D'Angelo[59]	High dose antithrombin supplementation vs placebo in early preeclampsia	Perinatal	Primary
2016	Nijman[60]	Nifedipine versus placebo in the treatment of preterm pre-labor rupture of membranes (APOSTEL IV)	Perinatal	Primary
2016	Gyamfi-Bannerman[61]	Antenatal betamethasone vs placebo for women at risk for late preterm delivery	Perinatal	Primary
2016	Elden[62]	protocol of SWEPIS- to compare induction of labour at 41 completed gestational weeks versus expectant management and induction at 42 completed gestational weeks	Perinatal	Primary

Supplementary Data 2: Table of Included Studies – (with references)

2016	Fang[63]	Effects of quercetin and melatonin in pregnant and gestational diabetic women	Perinatal	Primary
2016	Garcia[64]	Do knowledge of uterine artery resistance in the second trimester and targeted surveillance improve maternal and perinatal outcome? UTOPIA study	1. Maternal - General<br>2. Combined	Primary and Secondary
2016	Henrichs[65]	Effectiveness and cost-effectiveness of routine third trimester ultrasound screening for intrauterine growth restriction: protocol (The IRIS Study)	1. Maternal - General<br>2. Maternal - General<br>3. Perinatal	Primary and Secondary
2016	Tita[66]	Adjunctive azithromycin prophylaxis vs placebo for cesarean delivery	1. Maternal - General<br>2. Maternal - General<br>3. Perinatal<br>4. Perinatal	Primary and Secondary
2016	Widmer[67]	Room temperature stable carbetocin vs oxytocin for the prevention of postpartum haemorrhage during the third stage of labour in women delivering vaginally: protocol	Maternal - General	Primary and Secondary
2016	Kakuru[68]	Dihydroartemisinin-piperaquine for the prevention of malaria in pregnancy	Combined	Secondary
2016	Nicolaides[69]	Cervical pessary placement for prevention of preterm birth in unselected twin pregnancies:	Perinatal	Secondary

Supplementary Data 2: Table of Included Studies – (with references)

2016	Hezelgrave[70]	design of SuPPoRT:to compare three treatments: Cervical cerclage, cervical pessary and vaginal progesterone, for the prevention of preterm birth in women who develop a short cervix	Perinatal	Secondary
2016	Hermans[71]	Effectiveness of a cervical pessary for women who did t deliver 48 h after threatened preterm labor (Apostel VI)	Perinatal	Secondary
2016	Goya[72]	Cervical pessary vs expectant management to prevent preterm birth in women with twin gestation and so graphic short cervix: A multicenter (PECEP-Twins)	Perinatal	Secondary
2016	Morris[73]	Immediate delivery compared with expectant management after preterm pre-labour rupture of the membranes close to term (PPROMT trial):	Perinatal	Secondary
2016	Connolly[74]	A randomized trial of Foley balloon induction of labor trial in nulliparas (FIAT-N)	Perinatal	Secondary
2015	Equy[75]	Clinical impact of the disposable ventouse iCup versus a metallic vacuum cup	Combined	Primary
2015	Broekhuijsen[76]	Immediate delivery versus expectant monitoring for hypertensive disorders of pregnancy between 34	Maternal - General	Primary

Supplementary Data 2: Table of Included Studies – (with references)

		and 37 weeks of gestation (HYPITAT-II)		
2015	Boulvain[77]	Induction of labour versus expectant management for large-for-date fetuses	Perinatal	Primary
2015	Van Os[78]	Preventing Preterm Birth with Progesterone in Women with a Short Cervical Length from a Low-Risk Population	Perinatal	Primary
2015	Hammad[79]	Uncomplicated Pregnancies- third trimester ultrasonographic exams versus routine prenatal care) to improve the detection of small for gestational age	Perinatal	Primary
2015	George[80]	Comparison of neonatal outcomes in women with gestational diabetes with moderate hyperglycaemia on metformin or glibenclamide	Perinatal	Primary
2015	Awwad[81]	17-hydroxyprogesterone caproate vs placebo for the prevention of preterm birth in twin gestation (PROGESTWIN)	Perinatal	Primary
2015	Belfort[82]	"open" or "masked" monitoring with fetal ST-segment analysis	Perinatal	Primary

Supplementary Data 2: Table of Included Studies – (with references)

2015	Desai[83]	Intermittent screening and treatment or intermittent preventive treatment with dihydroartemisinin-piperaquine versus intermittent preventive treatment with sulfadoxine-pyrimethamine for the control of malaria during pregnancy in western Kenya	Perinatal	Primary and Secondary
2015	Cluver[84]	Double blind, randomised, placebo controlled trial to evaluate the efficacy of esomeprazole to treat early onset pre-eclampsia (PIE Trial): A protocol	1. Maternal - General<br>2. Perinatal<br>3. Perinatal	Secondary
2015	Combs[85]	17-hydroxyprogesterone caproate vs placebo for preterm rupture of the membranes	Perinatal	Secondary
2015	Brizot[86]	Vaginal progesterone vs placebo for the prevention of preterm birth in twin gestations	Perinatal	Secondary
2014	Alves[87]	BRAMAG trial: Oral magnesium supplementation in pregnancy for the prevention of preterm birth and perinatal and maternal morbidity	1. Maternal - General<br>2. Perinatal	Primary
2014	Rodger[88]	Antepartum dalteparin versus no antepartum dalteparin for the prevention of pregnancy complications in pregnant women with thrombophilia (TIPPS)	Combined	Primary

Supplementary Data 2: Table of Included Studies – (with references)

2014	McClure[89]	ultrasound to improve pregnancy outcomes in low income country settings	Combined	Primary
2014	MacKeen[90]	Suture compared with staple skin closure after cesarean delivery	Maternal - Wound	Primary
2014	Esmer[91]	subcutaneous closure vs non-subcut closure in preventing wound complications after cesarean delivery with Pfannenstiel incision:	Maternal - Wound	Primary
2014	Liem[92]	Cervical pessaries for prevention of preterm birth in women with a multiple pregnancy (ProTWIN): , open-label	Perinatal	Primary
2014	Barrett[93]	planned cesarean or vaginal delivery for twin pregnancy	Perinatal	Primary
2014	Schmitz[94]	Outpatient cervical ripening by nitric oxide donors vs placebo for prolonged pregnancy	Perinatal	Primary
2014	Slaghekke[95]	Fetoscopic laser coagulation of the vascular equator versus selective coagulation for twin-to-twin transfusion syndrome	Perinatal	Primary
2014	Kamat[96]	Comparison of nifedipine and progesterone for maintenance tocolysis after arrested preterm labour	Perinatal	Primary

Supplementary Data 2: Table of Included Studies – (with references)

2014	Galyean[97]	Removal versus retention of cerclage in preterm premature rupture of membranes	Perinatal	Secondary
2013	Ayala[98]	Chronotherapy with low-dose aspirin for prevention of complications in pregnancy	Combined	Primary
2013	Glavind[99]	Elective caesarean section at 38 weeks versus 39 weeks: Neonatal and maternal outcomes	Maternal - General	Primary
2013	CORONIS Collaborative Group (Brocklehurst)[100]	Caesarean section surgical techniques (CORONIS)	Maternal - General	Primary
2013	Figueroa[101]	Surgical staples compared with subcuticular suture for skin closure after cesarean delivery	Maternal - Wound	Primary
2013	Francis[102]	Timing of prophylactic antibiotic at cesarean section: A double-blinded, randomized trial	Maternal - Wound	Primary
2013	Roos[103]	effect of maintenance tocolysis with nifedipine vs placebo in threatened preterm labor on perinatal outcomes	Perinatal	Primary
2013	Heazell[104]	standard or intensive management of reduced fetal movements after 36 weeks gestation-feasibility study	Perinatal	Primary

Supplementary Data 2: Table of Included Studies – (with references)

2013	Kwak[105]	The efficacy of cefazolin plus macrolide (erythromycin or clarithromycin) versus cefazolin alone in neonatal morbidity and placental inflammation for women with PPROM	Perinatal	Primary
2013	Nuthalapaty[106]	A RCT of early versus delayed skin staple removal following caesarean section in the obese patient	Maternal - Wound	Secondary
2013	Crowther[107]	Magnesium sulphate at 30 to 34 weeks' gestational age vs placebo: Neuroprotection trial (MAGENTA) - protocol	Perinatal	Secondary
2013	Vigil-De Gracia[108]	steroids with prompt delivery after 48 hours vs steroids and expectant managements for severe preeclampsia remote from term: The MEXPRE Latin Study,	Perinatal	Secondary
2012	Seal[109]	Cesarean or vaginal delivery on maternal and perinatal outcome in women with eclampsia? A randomized controlled pilot study	1. Maternal - General<br>2. Perinatal	Primary
2012	Martinelli[110]	Heparin vs medical surveillance alone in pregnant women with previous placenta-mediated pregnancy complications	Combined	Primary

Supplementary Data 2: Table of Included Studies – (with references)

2012	Dioulasso[111]	Maternal HIV-1 disease progression 18-24 months postdelivery according to antiretroviral prophylaxis regimen (triple-antiretroviral prophylaxis during pregnancy and breastfeeding vs zidovudine/single-dose nevirapine prophylaxis):kesho bora	Maternal - General	Primary
2012	Grobman[112]	17 alpha-hydroxyprogesterone caproate vs placebo to prevent prematurity in nulliparas with cervical length less than 30 mm	Perinatal	Primary
2012	Dodd[113]	Elective birth at 37 weeks of gestation versus standard care for women with an uncomplicated twin pregnancy at term: The Twins Timing of Birth Trial	Perinatal	Primary
2012	Norman[114]	Trial protocol OPPTIMUM- Does progesterone prophylaxis for the prevention of preterm labour improve outcome?	Perinatal	Primary and Secondary
2011	Gris[115]	Addition of enoxaparin to aspirin for the Secondary prevention of placental vascular complications in women with severe pre-eclampsia: NOH-PE trial	Combined	Primary
2011	Gris[116]	enoxaparin for the Secondary prevention of placental vascular complications in women with previous severe pre-eclampsia: NOH-PE study	Combined	Primary

Supplementary Data 2: Table of Included Studies – (with references)

2011	Scifres[117]	Supplemental oxygen (nasal cannula vs non-rebreathe) for the prevention of post cesarean infectious morbidity	Maternal - General	Primary
2011	Boers[118]	Induction versus expectant monitoring for intrauterine growth restriction at term: Randomised equivalence trial (DIGITAT)	Perinatal	Primary
2011	Combs[119]	17-hydroxyprogesterone caproate vs placebo for twin pregnancy	Perinatal	Primary
2011	Lim[120]	17alpha-hydroxyprogesterone caproate vs placebo for the prevention of adverse neonatal outcome in multiple pregnancies:	Perinatal	Primary
2011	Combs[121]	17-Hydroxyprogesterone caproate vs placebo to prolong pregnancy after preterm rupture of the membranes	Perinatal	Primary
2010	Haas[122]	Vaginal cleansing vs. no cleaning before cesarean delivery to reduce postoperative infectious morbidity	Maternal - General	Primary
2010	Vanky[123]	Metformin Versus placebo from first trimester to delivery in polycystic ovary syndrome	Combined	Primary
2010	Basha[124]	wound complication rates of subcuticular suture vs staples for skin closure at cesarean delivery	Maternal - Wound	Primary

Supplementary Data 2: Table of Included Studies – (with references)

2010	Combs[125]	Failure of 17-hydroxyprogesterone to reduce neonatal morbidity or prolong triplet pregnancy	Perinatal	Primary
2010	Macones[126]	scaling and root planning (active) or tooth polishing (control) on occurrence of preterm birth: (PIPS)	1. Maternal - Wound<br>2. Perinatal	Secondary
2009	Nassar[127]	Two dose regimens of nifedipine for management of preterm labor	1. Maternal -General<br>2. Perinatal	Primary
2009	Landon[128]	usual prenatal care or dietary intervention, self-monitoring of BM, and insulin therapy, if necessary for mild gestational diabetes	Combined	Primary
2009	Rey[129]	Dalteparin vs. no dalteparin for the prevention of recurrence of placental-mediated complications of pregnancy in women without thrombophilia	Combined	Primary
2009	Koopmans[130]	Induction of labour versus expectant monitoring for gestational hypertension or mild pre-eclampsia after 36 weeks' gestation (HYPITAT):	Maternal - General	Primary
2009	Garite[131]	Impact of a 'rescue course' of antenatal corticosteroids vs placebo on neonatal morbidity	Perinatal	Primary
2009	Roos[132]	perinatal outcome after sustained tocolysis in early labour (APOSTEL-II trial)	Perinatal	Primary

Supplementary Data 2: Table of Included Studies – (with references)

2009	Caritis[133]	Prevention of preterm birth in triplets using 17 alpha-hydroxyprogesterone caproate	1. Perinatal<br>2. Combined	Primary and Secondary
2008	Rouse[134]	magnesium sulfate for the prevention of cerebral palsy	Perinatal	Primary
2008	Mazumder[135]	Single versus multiple courses of antenatal betamethasone and neonatal outcome	Perinatal	Primary
2008	Murphy[136]	Multiple courses of antenatal corticosteroids vs placebo for preterm birth (MACS)	Perinatal	Primary
2008	Rowan[137]	Metformin versus insulin for the treatment of gestational diabetes	Perinatal	Primary
2008	Nicholson[138]	The impact of the active management of risk in pregnancy at term on birth outcomes	Perinatal	Primary
2007	Marret[139]	Magnesium sulphate given before very-preterm birth to protect infant brain: PREMAG trial	Combined	Primary
2007	Smith[140]	placebo vs transdermal nitroglycerin for preterm labor	Perinatal	Primary
2006	Wapner[141]	Single versus weekly courses of antenatal corticosteroids: Evaluation of safety and efficacy	Perinatal	Primary
2006	Ovalle[142]	Antibiotic administration to patients with preterm labor and intact	Perinatal	Primary

		membranes in patients with endocervical inflammation		
2005	Fraser[143]	Amnioinfusion for the prevention of the meconium aspiration syndrome	Combined	Primary
2005	Ramsey[144]	Subcutaneous tissue reapproximation, alone or in combination with drain, in obese women undergoing cesarean delivery	Maternal - Wound	Primary
2005	Ganzevoort[145]	two temporising management strategies, one with and one without plasma volume expansion, for severe and early onset pre-eclampsia	Perinatal	Primary
2003	Harrigill[146]	intraabdominal irrigation vs no irrigation at cesarean delivery on maternal morbidity	Maternal - General	Primary
2003	Crowther[147]	Effect of Magnesium Sulfate Given for Neuroprotection before Preterm Birth	Perinatal	Primary and Secondary
2003	Segel[148]	Duration of antibiotic therapy (3 vs 7 days) after preterm premature rupture of fetal membranes	Perinatal	Secondary
2002	Landon[149]	diet therapy and insulin as required versus specific treatment for mild gestational diabetes mellitus	Combined	Primary

Supplementary Data 2: Table of Included Studies – (with references)

2001	Kenyon[150]	ORACLE - Antibiotics for preterm prelabour rupture of the membranes: Short-term and long-term outcomes	Perinatal	Primary
2001	Guinn[151]	Single vs weekly courses of antenatal corticosteroids for women at risk of preterm delivery	Perinatal	Primary
2001	Kenyon[152]	Broad-spectrum antibiotics for spontaneous preterm labour (ORACLE II)	Perinatal	Primary
2001	Kenyon[153]	Broad-spectrum antibiotics for preterm, prelabour rupture of fetal membranes (ORACLE I)	Perinatal	Primary
2000	Hofmeyer[154]	Planned caesarean section versus planned vaginal birth for breech presentation at term: a randomised multicentre trial.	1. Maternal - General<br>2. Perinatal	Primary and Secondary
1997	Rouse[155]	Chlorhexidine vs sterile water vaginal irrigation for the prevention of peripartal infection	Maternal - General	Primary
1997	Mercer[156]	Antibiotic therapy for reduction of infant morbidity after preterm premature rupture of the membranes	Perinatal	Primary

Supplementary Data 2: Table of Included Studies – (with references)

References

1. Pels A, Jakobsen JC, Ganzevoort W, et al. Detailed statistical analysis plan for the Dutch STRIDER (Sildenafil TheRapy in Dismal prognosis Early-onset fetal growth Restriction) randomised clinical trial on sildenafil versus placebo for pregnant women with severe early onset fetal growth restriction. Trials 2019;20:42.
2. Pasquier JC, Claris O, Rabilloud M, et al. Intentional early delivery versus expectant management for preterm premature rupture of membranes at 28-32 weeks' gestation: A multicentre randomized controlled trial (MICADO STUDY). European Journal of Obstetrics Gynecology and Reproductive Biology 2019;233:30-7.
3. Baqui AH, Lee ACC, Koffi AK, et al. Prevalence of and risk factors for abnormal vaginal flora and its association with adverse pregnancy outcomes in a rural district in north-east Bangladesh. Acta Obstetricia et Gynecologica Scandinavica 2018.
4. Simon VB, Fong A, Nageotte MP. Supplemental Oxygen Study: A Randomized Controlled Study on the Effect of Maternal Oxygen Supplementation during Planned Cesarean Delivery on Umbilical Cord Gases. American Journal of Perinatology 2018;35:84-9.
5. Simmons D, Hague WM, Teede HJ, et al. Hyperglycaemia in early pregnancy: the Treatment of Booking Gestational diabetes Mellitus (TOBOGM) study. A randomised controlled trial. The Medical journal of Australia 2018;209:405-6.
6. Venkateswaran M, Morkrid K, Ghanem B, et al. eRegQual-an electronic health registry with interactive checklists and clinical decision support for improving quality of antenatal care: Study protocol for a cluster randomized trial. Trials 2018;19:54.
7. Goldenberg RL, Nathan RO, Swanson D, et al. Routine antenatal ultrasound in low- and middle-income countries: first look - a cluster randomised trial. BJOG: An International Journal of Obstetrics and Gynaecology 2018;125:1591-9.
8. Luitjes SHE, Hermens R, de Wit L, Heymans MW, van Tulder MW, Wouters M. An innovative implementation strategy to improve the use of Dutch guidelines on hypertensive disorders in pregnancy: A randomized controlled trial. Pregnancy Hypertens 2018;14:131-8.
9. Nathan HL, Duhig K, Vousden N, et al. Evaluation of a novel device for the management of high blood pressure and shock in pregnancy in low-resource settings: Study protocol for a stepped-wedge cluster-randomised controlled trial (CRADLE-3 trial). Trials 2018;19:206.
10. Zaki MN, Wing DA, McNulty JA. Comparison of staples vs subcuticular suture in class III obese women undergoing cesarean: a randomized controlled trial. Am J Obstet Gynecol 2018;218:451 e1- e8.
11. Scrafford JD, Reddy B, Rivard C, Vogel RI. Effect of intra-operative glove changing during cesarean section on post-operative complications: a randomized controlled trial. Arch Gynecol Obstet 2018;297:1449-54.

Supplementary Data 2: Table of Included Studies – (with references)

12. Armstrong-Buisseret L, Mitchell E, Hepburn T, et al. Reduced fetal movement intervention Trial-2 (ReMIT-2): Protocol for a pilot randomised controlled trial of standard care informed by the result of a placental growth factor (PlGF) blood test versus standard care alone in women presenting with reduced fetal movement at or after 36+ 0 weeks gestation 11 Medical and Health Sciences 1114 Paediatrics and Reproductive Medicine. Trials 2018;19:531.
13. Berry DC, Thomas SD, Dorman KF, et al. Rationale, design, and methods for the Medical Optimization and Management of Pregnancies with Overt Type 2 Diabetes (MOMPOD) study. BMC Pregnancy and Childbirth 2018;18:488.
14. Norman JE, Norrie J, Maclennan G, et al. Open randomised trial of the (Arabin) pessary to prevent preterm birth in twin pregnancy with health economics and acceptability: STOPPIT-2-a study protocol. BMJ Open 2018;8:e026430.
15. Chappell LC, Chambers J, Dixon PH, et al. Ursodeoxycholic acid versus placebo in the treatment of women with intrahepatic cholestasis of pregnancy (ICP) to improve perinatal outcomes: Protocol for a randomised controlled trial (PITCHES). Trials 2018;19:657.
16. Subtil D, Brabant G, Tilloy E, et al. Early clindamycin for bacterial vaginosis in pregnancy (PREMEVA): a multicentre, double-blind, randomised controlled trial. The Lancet 2018;392:2171-9.
17. Brocklehurst P, Field D, Greene K, et al. Computerised interpretation of the fetal heart rate during labour: A randomised controlled trial (INFANT). Health Technology Assessment 2018;22:1-218.
18. Hermans FJR, Schuit E, Bekker MN, et al. Cervical pessary after arrested preterm labor: A randomized controlled trial. Obstetrics and Gynecology 2018;132:741-9.
19. Cahill AG, Srinivas SK, Tita ATN, et al. Effect of Immediate vs Delayed Pushing on Rates of Spontaneous Vaginal Delivery among Nulliparous Women Receiving Neuraxial Analgesia: A Randomized Clinical Trial. JAMA - Journal of the American Medical Association 2018;320:1444-54.
20. Dugoff L, Berghella V, Sehdev H, Mackeen AD, Goetzl L, Ludmir J. Prevention of preterm birth with pessary in singletons (PoPPS): randomized controlled trial. Ultrasound in obstetrics & gynecology : the official journal of the International Society of Ultrasound in Obstetrics and Gynecology 2018;51:573-9.
21. Blackwell SC, Gyamfi-Bannerman C, Biggio JR, et al. PROLONG Clinical Study Protocol: Hydroxyprogesterone Caproate to Reduce Recurrent Preterm Birth. American Journal of Perinatology 2018;35:1228-34.
22. Grobman W. A randomized trial of elective induction of labor at 39 weeks compared with expectant management of low-risk nulliparous women. American Journal of Obstetrics and Gynecology 2018;218:S601.
23. Senat MV, Affres H, Letourneau A, et al. Effect of Glyburide vs subcutaneous insulin on perinatal complications among women with gestational diabetes a randomized clinical trial. JAMA - Journal of the American Medical Association 2018;319:1773-80.
24. Balogun OA, Sibai BM, Pedroza C, Blackwell SC, Barrett TL, Chauhan SP. Serial Third-Trimester Ultrasonography Compared With Routine Care in Uncomplicated Pregnancies: A Randomized Controlled Trial. Obstetrics and gynecology 2018;132:1358-67.

Supplementary Data 2: Table of Included Studies – (with references)

25. Norman JE, Marlow N, Messow CM, et al. Does progesterone prophylaxis to prevent preterm labour improve outcome? A randomised double-blind placebo-controlled trial (OPPTIMUM). Health Technology Assessment 2018;22:1-304.
26. Price S, Nankervis A, Permezel M, Prendergast L, Sumithran P, Proietto J. Health consequences for mother and baby of substantial pre-conception weight loss in obese women: Study protocol for a randomized controlled trial. Trials 2018;19:248.
27. Wihbey KA, Joyce EM, Spalding ZT, et al. Prophylactic Negative Pressure Wound Therapy and Wound Complication after Cesarean Delivery in Women with Class II or III Obesity: A Randomized Controlled Trial. Obstetrics and Gynecology 2018;132:377-84.
28. Bardou M, Crepon B, Bertaux AC, et al. NAITRE study on the impact of conditional cash transfer on poor pregnancy outcomes in underprivileged women: Protocol for a nationwide pragmatic cluster-randomised superiority clinical trial in France. BMJ Open 2017;7:017321.
29. Schreiber K, Breen K, Cohen H, et al. HYdroxychloroquine to Improve Pregnancy Outcome in Women with AnTIphospholipid Antibodies (HYPATIA) Protocol: A Multinational Randomized Controlled Trial of Hydroxychloroquine versus Placebo in Addition to Standard Treatment in Pregnant Women with Antiphospholipid Syndrome or Antibodies. Seminars in Thrombosis and Hemostasis 2017;43:562-71.
30. Fransen AF, van de Ven J, Schuit E, van Tetering A, Mol BW, Oei SG. Simulation-based team training for multi-professional obstetric care teams to improve patient outcome: a multicentre, cluster randomised controlled trial. BJOG 2017;124:641-50.
31. Linden JA, Grimmnitz B, Hagopian L, et al. Is the Pelvic Examination Still Crucial in Patients Presenting to the Emergency Department With Vaginal Bleeding or Abdominal Pain When an Intrauterine Pregnancy Is Identified on Ultrasonography? A Randomized Controlled Trial. Ann Emerg Med 2017;70:825-34.
32. Ruhstaller K, Downes KL, Chandrasekaran S, Srinivas S, Durnwald C. Prophylactic Wound Vacuum Therapy after Cesarean Section to Prevent Wound Complications in the Obese Population: A Randomized Controlled Trial (the ProVac Study). Am J Perinatol 2017;34:1125-30.
33. Pasquier JC, Fraser W, Blouin S, et al. A cluster-randomized trial to reduce major perinatal morbidity among women with one prior cesarean delivery in Quebec (PRISMA trial): Study protocol for a randomized controlled trial. Trials 2017;18:434.
34. Zijl MDv, Koullali B, Naaktgeboren CA, et al. Pessary or Progesterone to Prevent Preterm delivery in women with short cervical length: The Quadruple P randomised controlled trial. BMC Pregnancy and Childbirth 2017;17:284.
35. Berghella V, Dugoff L, Ludmir J. Prevention of preterm birth with pessary in twins (PoPPT): a randomized controlled trial. Ultrasound in obstetrics & gynecology : the official journal of the International Society of Ultrasound in Obstetrics and Gynecology 2017;49:567-72.

Supplementary Data 2: Table of Included Studies – (with references)

36. Dodd J, Andersen C, Dickinson J, Kilby M, Windrim R, Ryan G. Fetal middle cerebral artery Doppler to time second and subsequent intrauterine transfusions to treat anaemia due to red cell allo-immunisation: A randomised trial. BJOG: An International Journal of Obstetrics and Gynaecology 2017;124:11-2.
37. Semrau KEA, Hirschhorn LR, Delaney MM, et al. Outcomes of a coaching-based WHO safe childbirth checklist program in India. New England Journal of Medicine 2017;377:2313-24.
38. Groom KM, McCowan LM, Mackay LK, et al. Enoxaparin for the prevention of preeclampsia and intrauterine growth restriction in women with a history: a randomized trial. American Journal of Obstetrics and Gynecology 2017;216:296.
39. Springel EH, Wang XY, Sarfoh V, Stetzer B, Weight S, Mercer B. A randomized controlled trial of chlorhexidine-alcohol versus povidone-iodine for cesarean antisepsis. American Journal of Obstetrics and Gynecology 2017;216:S30.
40. Koullali B, Kempen LEMv, Zijl MDv, et al. A multi-centre, non-inferiority, randomised controlled trial to compare a cervical pessary with a cervical cerclage in the prevention of preterm delivery in women with short cervical length and a history of preterm birth - PC study. BMC Pregnancy and Childbirth 2017;17:215.
41. Rodo C, Arevalo S, Lewi L, et al. Arabin cervical pessary for prevention of preterm birth in cases of twin-to-twin transfusion syndrome treated by fetoscopic LASER coagulation: The PECEP LASER randomised controlled trial. BMC Pregnancy and Childbirth 2017;17:256.
42. Visser L, Boer MAd, Groot CJMd, et al. Low dose aspirin in the prevention of recurrent spontaneous preterm labour - the APRIL study: A multicenter randomized placebo controlled trial. BMC Pregnancy and Childbirth 2017;17:223.
43. Crowther CA, Ashwood P, McPhee AJ, et al. Vaginal progesterone pessaries for pregnant women with a previous preterm birth to prevent neonatal respiratory distress syndrome (the PROGRESS Study): A multicentre, randomised, placebo-controlled trial. PLoS Medicine 2017;14:e1002390.
44. Connolly KA, Kohari KS, Factor SH, et al. A Randomized Trial of Foley Balloon Induction of Labor Trial in Multiparas (FIAT-M). American Journal of Perinatology 2017;34:1108-14.
45. Wattar BHA, Dodds J, Placzek A, et al. Effect of simple, targeted diet in pregnant women with metabolic risk factors on maternal and fetal outcomes (ESTEEM): Study protocol for a pragmatic multicentre randomised trial. BMJ Open 2016;6:e013495.
46. Barasinski C, Vendittelli F. Effect of the type of maternal pushing during the second stage of labour on obstetric and neonatal outcome: A multicentre randomised trial - The EOLE study protocol. BMJ Open 2016;6:e012290.
47. Salim R, Nachum Z, Gavish I, Romano S, Braverman M, Garmi G. Adjusting enoxaparin dosage according to anti-fxa levels and pregnancy outcome in thrombophilic women: A randomised controlled trial. Thrombosis and Haemostasis 2016;116:687-95.
48. Feig DS, Murphy K, Asztalos E, et al. Metformin in women with type 2 diabetes in pregnancy (MiTy): A multi-center randomized controlled trial. BMC Pregnancy and Childbirth 2016;16:173.

Supplementary Data 2: Table of Included Studies – (with references)

49. Kimani J, Phiri K, Kamiza S, et al. Efficacy and safety of azithromycin-chloroquine versus sulfadoxine-pyrimethamine for intermittent preventive treatment of plasmodium falciparum malaria infection in pregnant women in Africa: An open-label, randomized trial. PLoS ONE 2016;11:e0157045.
50. Haddad B, Winer N, Chitrit Y, et al. Enoxaparin and Aspirin Compared with Aspirin Alone to Prevent Placenta-Mediated Pregnancy Complications. Obstetrics and Gynecology 2016;128:1053-63.
51. Madanitsa M, Kalilani L, Mwapasa V, et al. Scheduled Intermittent Screening with Rapid Diagnostic Tests and Treatment with Dihydroartemisinin-Piperaquine versus Intermittent Preventive Therapy with Sulfadoxine-Pyrimethamine for Malaria in Pregnancy in Malawi: An Open-Label Randomized Controlled Trial. PLoS Medicine 2016;13:e1002124.
52. McCarthy EA, Walker SP, Ugoni A, Lappas M, Leong O, Shub A. Self-weighing and simple dietary advice for overweight and obese pregnant women to reduce obstetric complications without impact on quality of life: A randomised controlled trial. BJOG: An International Journal of Obstetrics and Gynaecology 2016;123:965-73.
53. Ducloy-Bouthors AS, Mignon A, Huissoud C, Grouin JM, Mercier FJ. Fibrinogen concentrate as a treatment for postpartum haemorrhage-induced coagulopathy: A study protocol for a randomised multicentre controlled trial. The fibrinogen in haemorrhage of DELivery (FIDEL) trial. Anaesthesia Critical Care and Pain Medicine 2016;35:293-8.
54. Duggal N, Poddatorri V, Noroozkhani S, Siddik-Ahmad RI, Caughey AB. Perioperative oxygen supplementation and surgical site infection after cesarean delivery: A randomized trial. Obstetrics and Gynecology 2013;122:79-84.
55. Vliet EOGV, Nijman TAJ, Schuit E, et al. Nifedipine versus atosiban for threatened preterm birth (APOSTEL III): A multicentre, randomised controlled trial. The Lancet 2016;387:2117-24.
56. Norman JE, Marlow N, Messow CM, et al. Vaginal progesterone prophylaxis for preterm birth (the OPPTIMUM study): A multicentre, randomised, double-blind trial. The Lancet 2016;387:2106-16.
57. Eikelder MLGT, Rengerink KO, Jozwiak M, et al. Induction of labour at term with oral misoprostol versus a Foley catheter (PROBAAT-II): A multicentre randomised controlled non-inferiority trial. The Lancet 2016;387:1619-28.
58. Murphy VE, Jensen ME, Mattes J, et al. The Breathing for Life Trial: A randomised controlled trial of fractional exhaled nitric oxide (FENO)-based management of asthma during pregnancy and its impact on perinatal outcomes and infant and childhood respiratory health. BMC Pregnancy and Childbirth 2016;16:111.
59. Angelo AD, Valsecchi L. High dose antithrombin supplementation in early preeclampsia: A randomized, double blind, placebo-controlled study. Thrombosis Research 2016;140:7-13.
60. Nijman TAJ, Vliet EOGv, Naaktgeboren CA, et al. Nifedipine versus placebo in the treatment of preterm prelabor rupture of membranes: a randomized controlled trial: Assessment of perinatal outcome by use of tocolysis in early labor-APOSTEL IV trial. European Journal of Obstetrics Gynecology and Reproductive Biology 2016;205:79-84.
61. Gyamfi-Bannerman C, Thom EA, Blackwell SC, et al. Antenatal Betamethasone for Women at Risk for Late Preterm Delivery. N Engl J Med 2016;374:1311-20.

Supplementary Data 2: Table of Included Studies – (with references)

62. Elden H, Hagberg H, Wessberg A, et al. Study protocol of SWEPIS a Swedish multicentre register based randomised controlled trial to compare induction of labour at 41 completed gestational weeks versus expectant management and induction at 42 completed gestational weeks. BMC Pregnancy and Childbirth 2016;16:49.
63. Fang JH, Zhang SH, Yu XM, Yang Y. Effects of quercetin and melatonin in pregnant and gestational diabetic women. Latin American Journal of Pharmacy 2016;35:1420-5.
64. Garcia B, Llurba E, Valle L, et al. Do knowledge of uterine artery resistance in the second trimester and targeted surveillance improve maternal and perinatal outcome? UTOPIA study: a randomized controlled trial. Ultrasound in obstetrics & gynecology : the official journal of the International Society of Ultrasound in Obstetrics and Gynecology 2016;47:680-9.
65. Henrichs J, Verfaille V, Viester L, et al. Effectiveness and cost-effectiveness of routine third trimester ultrasound screening for intrauterine growth restriction: Study protocol of a nationwide stepped wedge cluster-randomized trial in The Netherlands (The IRIS Study). BMC Pregnancy and Childbirth 2016;16:310.
66. Tita ATN, Szychowski JM, Boggess K, et al. Adjunctive azithromycin prophylaxis for cesarean delivery. New England Journal of Medicine 2016;375:1231-41.
67. Widmer M, Piaggio G, Abdel-Aleem H, et al. Room temperature stable carbetocin for the prevention of postpartum haemorrhage during the third stage of labour in women delivering vaginally: Study protocol for a randomized controlled trial. Trials 2016;17:143.
68. Kakuru A, Jagannathan P, Muhindo MK, et al. Dihydroartemisinin-piperaquine for the prevention of malaria in pregnancy. New England Journal of Medicine 2016;374:928-39.
69. Nicolaides KH, Syngelaki A, Poon LC, et al. Cervical pessary placement for prevention of preterm birth in unselected twin pregnancies: A randomized controlled trial. American Journal of Obstetrics and Gynecology 2016;214:3.
70. Hezelgrave NL, Watson HA, Ridout A, et al. Rationale and design of SuPPoRT: A multi-centre randomised controlled trial to compare three treatments: Cervical cerclage, cervical pessary and vaginal progesterone, for the prevention of preterm birth in women who develop a short cervix. BMC Pregnancy and Childbirth 2016;16:358.
71. Hermans FJR, Schuit E, Opmeer BC, et al. Effectiveness of a cervical pessary for women who did not deliver 48 h after threatened preterm labor (Assessment of perinatal outcome after specific treatment in early labor: Apostel VI trial). BMC Pregnancy and Childbirth 2016;16:154.
72. Goya M, Calle MDL, Pratcorona L, et al. Cervical pessary to prevent preterm birth in women with twin gestation and sonographic short cervix: A multicenter randomized controlled trial (PECEP-Twins). American Journal of Obstetrics and Gynecology 2016;214:145-52.
73. Morris JM, Roberts CL, Bowen JR, et al. Immediate delivery compared with expectant management after preterm pre-labour rupture of the membranes close to term (PPROMT trial): A randomised controlled trial. The Lancet 2016;387:444-52.

Supplementary Data 2: Table of Included Studies – (with references)

74. Connolly KA, Kohari KS, Rekawek P, et al. A randomized trial of Foley balloon induction of labor trial in nulliparas (FIAT-N). American Journal of Obstetrics and Gynecology 2016;215:392.
75. Equy V, David-Tchouda S, Dreyfus M, et al. Clinical impact of the disposable ventouse iCup versus a metallic vacuum cup: A multicenter randomized controlled trial. BMC Pregnancy and Childbirth 2015;15:332.
76. Broekhuijsen K, Baaren GJV, Pampus MGV, et al. Immediate delivery versus expectant monitoring for hypertensive disorders of pregnancy between 34 and 37 weeks of gestation (HYPITAT-II): An open-label, randomised controlled trial. The Lancet 2015;385:2492-501.
77. Boulvain M, Senat MV, Perrotin F, et al. Induction of labour versus expectant management for large-for-date fetuses: A randomised controlled trial. The Lancet 2015;385:2600-5.
78. Os MAV, Ven AJVD, Kleinrouweler CE, et al. Preventing Preterm Birth with Progesterone in Women with a Short Cervical Length from a Low-Risk Population: A Multicenter Double-Blind Placebo-Controlled Randomized Trial. American Journal of Perinatology 2015;32:993-1000.
79. Hammad IA, Chauhan SP, Mlynarczyk M, et al. Uncomplicated Pregnancies and Ultrasounds for Fetal Growth Restriction: A Pilot Randomized Clinical Trial. AJP Reports 2015;6:e83-e90.
80. George A, Mathews JE, Sam D, et al. Comparison of neonatal outcomes in women with gestational diabetes with moderate hyperglycaemia on metformin or glibenclamide - A randomised controlled trial. Australian and New Zealand Journal of Obstetrics and Gynaecology 2015;55:47-52.
81. Awwad J, Usta IM, Ghazeeri G, et al. A randomised controlled double-blind clinical trial of 17-hydroxyprogesterone caproate for the prevention of preterm birth in twin gestation (PROGESTWIN): Evidence for reduced neonatal morbidity. BJOG: An International Journal of Obstetrics and Gynaecology 2015;122:71-9.
82. Belfort MA, Saade GR, Thom E, et al. A randomized trial of intrapartum fetal ECG ST-segment analysis. New England Journal of Medicine 2015;373:632-41.
83. Desai M, Gutman J, Lanziva AL, et al. Intermittent screening and treatment or intermittent preventive treatment with dihydroartemisinin-piperaquine versus intermittent preventive treatment with sulfadoxine-pyrimethamine for the control of malaria during pregnancy in western Kenya: An open-label, three-group, randomised controlled superiority trial. The Lancet 2015;386:2507-19.
84. Cluver CA, Walker SP, Mol BW, et al. Double blind, randomised, placebocontrolled trial to evaluate the efficacy of esomeprazole to treat early onset pre-eclampsia (PIE Trial): A study protocol. BMJ Open 2015;5:e008211.
85. Combs CA, Garite TJ, Maurel K, et al. 17-hydroxyprogesterone caproate for preterm rupture of the membranes: A multicenter, randomized, double-blind, placebo-controlled trial. American Journal of Obstetrics and Gynecology 2015;213:364e1-e12.

Supplementary Data 2: Table of Included Studies – (with references)

86. Brizot ML, Hernandez W, Liao AW, et al. Vaginal progesterone for the prevention of preterm birth in twin gestations: a randomized placebo-controlled double-blind study. American Journal of Obstetrics and Gynecology 2015;213:82.
87. Alves JGB, Araujo CAFLd, Pontes IEA, Guimaraes AC, Ray JG. The BRAzil MAGnesium (BRAMAG) trial: A randomized clinical trial of oral magnesium supplementation in pregnancy for the prevention of preterm birth and perinatal and maternal morbidity. BMC Pregnancy and Childbirth 2014;14:222.
88. Rodger MA, Hague WM, Kingdom J, et al. Antepartum dalteparin versus no antepartum dalteparin for the prevention of pregnancy complications in pregnant women with thrombophilia (TIPPS): A multinational open-label randomised trial. The Lancet 2014;384:1673-83.
89. McClure EM, Nathan RO, Saleem S, et al. First look: A cluster-randomized trial of ultrasound to improve pregnancy outcomes in low income country settings. BMC Pregnancy and Childbirth 2014;14:73.
90. MacKeen AD, Khalifeh A, Fleisher J, et al. Suture compared with staple skin closure after cesarean delivery: A randomized controlled trial. Obstetrics and Gynecology 2014;123:1169-75.
91. Esmer AC, Goksedef PC, Akca A, et al. Role of subcutaneous closure in preventing wound complications after cesarean delivery with Pfannenstiel incision: A randomized clinical trial. Journal of Obstetrics and Gynaecology Research 2014;40:728-35.
92. Liem S, Schuit E, Hegeman M, et al. Cervical pessaries for prevention of preterm birth in women with a multiple pregnancy (ProTWIN): A multicentre, open-label randomised controlled trial. Obstetrical and Gynecological Survey 2014;69:73-5.
93. Barrett JFR, Hannah ME, Hutton EK, et al. Randomized trial of planned cesarean or vaginal delivery for twin pregnancy. Obstetrical and Gynecological Survey 2014;69:61-2.
94. Schmitz T, Fuchs F, Closset E, et al. Outpatient cervical ripening by nitric oxide donors for prolonged pregnancy. Obstetrics and Gynecology 2014;124:1089-97.
95. Slaghekke F, Lopriore E, Lewi L, et al. Fetoscopic laser coagulation of the vascular equator versus selective coagulation for twin-to-twin transfusion syndrome: An open-label randomised controlled trial. The Lancet 2014;383:2144-51.
96. Kamat S, Veena P, Rani R. Comparison of nifedipine and progesterone for maintenance tocolysis after arrested preterm labour. Journal of Obstetrics and Gynaecology 2014;34:322-5.
97. Galyean A, Garite TJ, Maurel K, et al. Removal versus retention of cerclage in preterm premature rupture of membranes: a randomized controlled trial. American journal of obstetrics and gynecology 2014;211:399-7.
98. Ayala DE, Ucieda R, Hermida RC. Chronotherapy with low-dose aspirin for prevention of complications in pregnancy. Chronobiology International 2013;30:260-79.

Supplementary Data 2: Table of Included Studies – (with references)

99. Glavind J, Kindberg SF, Uldbjerg N, et al. Elective caesarean section at 38 weeks versus 39 weeks: Neonatal and maternal outcomes in a randomised controlled trial. BJOG: An International Journal of Obstetrics and Gynaecology 2013;120:1123-32.
100. Group CC, Abalos E, Addo V, et al. Caesarean section surgical techniques (CORONIS): a fractional, factorial, unmasked, randomised controlled trial. Lancet 2013;382:234-48.
101. Figueroa D, Jauk VC, Szychowski JM, et al. Surgical staples compared with subcuticular suture for skin closure after cesarean delivery: A randomized controlled trial. Obstetrics and Gynecology 2013;121:33-8.
102. Francis C, Mumford M, Strand ML, Moore ES, Strand EA. Timing of prophylactic antibiotic at cesarean section: A double-blinded, randomized trial. Journal of Perinatology 2013;33:759-62.
103. Roos C, Spaanderman MEA, Schuit E, et al. Effect of maintenance tocolysis with nifedipine in threatened preterm labor on perinatal outcomes: A randomized controlled trial. JAMA - Journal of the American Medical Association 2013;309:41-7.
104. Heazell AEP, Bernatavicius G, Roberts SA, et al. A randomised controlled trial comparing standard or intensive management of reduced fetal movements after 36 weeks gestation-a feasibility study. BMC Pregnancy and Childbirth 2013;13:95.
105. Kwak HM, Shin MY, Cha HH, et al. The efficacy of cefazolin plus macrolide (erythromycin or clarithromycin) versus cefazolin alone in neonatal morbidity and placental inflammation for women with preterm premature rupture of membranes. Placenta 2013;34:346-52.
106. Nuthalapaty FS, Lee CM, Lee JH, Kuper SG, H. L. Higdon r. A randomized controlled trial of early versus delayed skin staple removal following caesarean section in the obese patient. Journal of obstetrics and gynaecology Canada : JOGC = Journal d'obstetrique et gynecologie du Canada : JOGC 2013;35:426-33.
107. Crowther CA, Middleton PF, Wilkinson D, Ashwood P, Haslam R. Magnesium sulphate at 30 to 34 weeks' gestational age: Neuroprotection trial (MAGENTA) - study protocol. BMC Pregnancy and Childbirth 2013;13:91.
108. Gracia PV-D, Tejada OR, Minaca AC, et al. Expectant management of severe preeclampsia remote from term: The MEXPRE Latin Study, a randomized, multicenter clinical trial. American Journal of Obstetrics and Gynecology 2013;209:425.
109. Seal SL, Ghosh D, Kamilya G, Mukherji J, Hazra A, Garain P. Does route of delivery affect maternal and perinatal outcome in women with eclampsia? A randomized controlled pilot study. American Journal of Obstetrics and Gynecology 2012;206:484.
110. Martinelli I, Ruggenenti P, Cetin I, et al. Heparin in pregnant women with previous placenta-mediated pregnancy complications: A prospective, randomized, multicenter, controlled clinical trial. Blood 2012;119:3269-75.
111. Dioulasso B, Faso B, Meda N, et al. Maternal HIV-1 disease progression 18-24 months postdelivery according to antiretroviral prophylaxis regimen (triple-antiretroviral prophylaxis during pregnancy and breastfeeding vs

zidovudine/single-dose nevirapine prophylaxis): The kesho bora randomized controlled trial. Clinical Infectious Diseases 2012;55:449-60.
112. Grobman WA, Thom EA, Spong CY, et al. 17 alpha-hydroxyprogesterone caproate to prevent prematurity in nulliparas with cervical length less than 30 mm. American Journal of Obstetrics and Gynecology 2012;207:390.
113. Dodd JM, Crowther CA, Haslam RR, Robinson JS. Elective birth at 37 weeks of gestation versus standard care for women with an uncomplicated twin pregnancy at term: The Twins Timing of Birth Randomised Trial. BJOG: An International Journal of Obstetrics and Gynaecology 2012;119:964-73.
114. Norman JE, Shennan A, Bennett P, et al. Trial protocol OPPTIMUM- Does progesterone prophylaxis for the prevention of preterm labour improve outcome? BMC Pregnancy and Childbirth 2012;12:79.
115. Gris JC, Chauleur C, Molinari N, et al. Addition of enoxaparin to aspirin for the secondary prevention of placental vascular complications in women with severe pre-eclampsia: The pilot randomised controlled NOH-PE trial. Thrombosis and Haemostasis 2011;106:1053-61.
116. Gris JCR, Chauleur C, Mares P, et al. Enoxaparin for the secondary prevention of placental vascular complications in women with previous severe pre-eclampsia: The pilot randomised controlled NOH-PE study. Journal of Thrombosis and Haemostasis 2011;9:753.
117. Scifres CM, Leighton BL, Fogertey PJ, MacOnes GA, Stamilio DM. Supplemental oxygen for the prevention of postcesarean infectious morbidity: A randomized controlled trial. American Journal of Obstetrics and Gynecology 2011;205:267.
118. Boers KE, Vijgen SMC, Bijlenga D, et al. Induction versus expectant monitoring for intrauterine growth restriction at term: Randomised equivalence trial (DIGITAT). BMJ 2011;342:35.
119. Combs CA, Garite T, Maurel K, Das A, Porto M. 17-hydroxyprogesterone caproate for twin pregnancy: A double-blind, randomized clinical trial. American Journal of Obstetrics and Gynecology 2011;204:221.
120. Lim AC, Schuit E, Bloemenkamp K, et al. 17alpha-hydroxyprogesterone caproate for the prevention of adverse neonatal outcome in multiple pregnancies: A randomized controlled trial. Obstetrics and Gynecology 2011;118:513-20.
121. Combs CA, Thomas JG, Kimberly M, et al. 17-Hydroxyprogesterone caproate to prolong pregnancy after preterm rupture of the membranes: early termination of a double-blind, randomized clinical trial. BMC research notes 2011;4:568.
122. Haas DM, Pazouki F, Smith RR, et al. Vaginal cleansing before cesarean delivery to reduce postoperative infectious morbidity: a randomized, controlled trial. American Journal of Obstetrics and Gynecology 2010;202:310.
123. Vanky E, Stridsklev S, Heimstad R, et al. Metformin Versus placebo from first trimester to delivery in polycystic ovary syndrome: A randomized, controlled multicenter study. Journal of Clinical Endocrinology and Metabolism 2010;95:E448-E55.

Supplementary Data 2: Table of Included Studies – (with references)

124. Basha SL, Rochon ML, Quiones JN, Coassolo KM, Rust OA, Smulian JC. Randomized controlled trial of wound complication rates of subcuticular suture vs staples for skin closure at cesarean delivery. American Journal of Obstetrics and Gynecology 2010;203:285.
125. Combs CA, Garite T, Maurel K, Das A, Porto M. Failure of 17-hydroxyprogesterone to reduce neonatal morbidity or prolong triplet pregnancy: A double-blind, randomized clinical trial. American Journal of Obstetrics and Gynecology 2010;203:248.
126. Macones GA, Parry S, Nelson DB, et al. Treatment of localized periodontal disease in pregnancy does not reduce the occurrence of preterm birth: results from the Periodontal Infections and Prematurity Study (PIPS). American Journal of Obstetrics and Gynecology 2010;202:147.
127. Nassar A, Abu-Musa AA, Awwad J, Khalil A, Tabbara J, Usta IM. Two dose regimens of nifedipine for management of preterm labor: A randomized controlled trial. American Journal of Perinatology 2009;26:575-81.
128. Landon MB, Spong CY, Thom E, et al. A multicenter, randomized trial of treatment for mild gestational diabetes. New England Journal of Medicine 2009;361:1339-48.
129. Rey E, Garneau P, David M, et al. Dalteparin for the prevention of recurrence of placental-mediated complications of pregnancy in women without thrombophilia: A pilot randomized controlled trial. Journal of Thrombosis and Haemostasis 2009;7:58-64.
130. Koopmans CM, Bijlenga D, Groen H, et al. Induction of labour versus expectant monitoring for gestational hypertension or mild pre-eclampsia after 36 weeks' gestation (HYPITAT): a multicentre, open-label randomised controlled trial. The Lancet 2009;374:979-88.
131. Garite TJ, Kurtzman J, Maurel K, Clark R. Impact of a 'rescue course' of antenatal corticosteroids: a multicenter randomized placebo-controlled trial. American Journal of Obstetrics and Gynecology 2009;200:248.
132. Roos C, Scheepers LHCJ, Bloemenkamp KWM, et al. Assessment of perinatal outcome after sustained tocolysis in early labour (APOSTEL-II trial). BMC Pregnancy and Childbirth 2009;9:42.
133. Caritis SN, Rouse DJ, Peaceman AM, et al. Prevention of preterm birth in triplets using 17 alpha-hydroxyprogesterone caproate: A randomized controlled trial. Obstetrics and Gynecology 2009;113:285-92.
134. Rouse DJ, Hirtz DG, Thom E, et al. A randomized, controlled trial of magnesium sulfate for the prevention of cerebral palsy. New England Journal of Medicine 2008;359:895-905.
135. Mazumder P, Dutta S, Kaur J, Narang A. Single versus multiple courses of antenatal betamethasone and neonatal outcome: A randomized controlled trial. Indian Pediatrics 2008;45:661-7.
136. Murphy KE, Hannah ME, Willan AR, et al. Multiple courses of antenatal corticosteroids for preterm birth (MACS): a randomised controlled trial. The Lancet 2008;372:2143-51.
137. Rowan JA, Hague WM, Gao W, Battin MR, Moore MP. Metformin versus insulin for the treatment of gestational diabetes. New England Journal of Medicine 2008;358:2003-15.

Supplementary Data 2: Table of Included Studies – (with references)

138. Nicholson JM, Parry S, Caughey AB, Rosen S, Keen A, Macones GA. The impact of the active management of risk in pregnancy at term on birth outcomes: a randomized clinical trial. American Journal of Obstetrics and Gynecology 2008;198:511.
139. Marret S, Marpeau L, Zupan-Simunek V, et al. Magnesium sulphate given before very-preterm birth to protect infant brain: The randomised controlled PREMAG trial. BJOG: An International Journal of Obstetrics and Gynaecology 2007;114:310-8.
140. Smith GN, Walker MC, Ohlsson A, Brien KO, Windrim R. Randomized double-blind placebo-controlled trial of transdermal nitroglycerin for preterm labor. American Journal of Obstetrics and Gynecology 2007;196:37.
141. Wapner RJ, Sorokin Y, Thom EA, et al. Single versus weekly courses of antenatal corticosteroids: Evaluation of safety and efficacy. American Journal of Obstetrics and Gynecology 2006;195:633-42.
142. Ovalle A, Romero R, Gomez R, et al. Antibiotic administration to patients with preterm labor and intact membranes: Is there a beneficial effect in patients with endocervical inflammation? Journal of Maternal-Fetal and Neonatal Medicine 2006;19:453-64.
143. Fraser WD, Hofmeyr J, Lede R, et al. Amnioinfusion for the prevention of the meconium aspiration syndrome. New England Journal of Medicine 2005;353:909-17.
144. Ramsey PS, White AM, Guinn DA, et al. Subcutaneous tissue reapproximation, alone or in combination with drain, in obese women undergoing cesarean delivery. Obstetrics and Gynecology 2005;105:967-73.
145. Ganzevoort W, Rep A, Bonsel GJ, et al. A randomised controlled trial comparing two temporising management strategies, one with and one without plasma volume expansion, for severe and early onset pre-eclampsia. BJOG: An International Journal of Obstetrics and Gynaecology 2005;112:1358-68.
146. Harrigill KM, Miller HS, Haynes DE. The effect of intraabdominal irrigation at cesarean delivery on maternal morbidity: A randomized trial. Obstetrics and Gynecology 2003;101:80-5.
147. Crowther CA, Hiller JE, Doyle LW, Haslam RR. Effect of Magnesium Sulfate Given for Neuroprotection before Preterm Birth: A Randomized Controlled Trial. Journal of the American Medical Association 2003;290:2669-76.
148. Segel SY, Miles AM, Clothier B, Parry S, Macones GA. Duration of antibiotic therapy after preterm premature rupture of fetal membranes. American Journal of Obstetrics and Gynecology 2003;189:799-802.
149. Landon MB, Thom E, Spong CY, et al. A planned randomized clinical trial of treatment for mild gestational diabetes mellitus. Journal of Maternal-Fetal and Neonatal Medicine 2002;11:226-31.
150. Kenyon S, Taylor DJ, Tarnow-Mordi WO. ORACLE - Antibiotics for preterm prelabour rupture of the membranes: Short-term and long-term outcomes. Acta Paediatrica, International Journal of Paediatrics, Supplement 2001;91:12-5.
151. Guinn DA, Atkinson MW, Sullivan L, et al. Single vs weekly courses of antenatal corticosteroids for women at risk of preterm delivery: A randomized controlled trial. Journal of the American Medical Association 2001;286:1581-7.

Supplementary Data 2: Table of Included Studies – (with references)

152. Kenyon SL, Taylor DJ, Tarnow-Mordi W, Oracle Collaborative G. Broad-spectrum antibiotics for spontaneous preterm labour: the ORACLE II randomised trial. ORACLE Collaborative Group. Lancet (London, England) 2001;357:989-94.
153. Kenyon SL, Taylor DJ, Tarnow-Mordi W, Oracle Collaborative G. Broad-spectrum antibiotics for preterm, prelabour rupture of fetal membranes: the ORACLE I randomised trial. ORACLE Collaborative Group. Lancet (London, England) 2001;357:979-88.
154. Hofmeyr GJ, Hannah M, Lawrie TA. Planned caesarean section for term breech delivery. The Cochrane database of systematic reviews 2015;7:CD000166.
155. Rouse DJ, Hauth JC, Andrews WW, Mills BB, Maher JE. Chlorhexidine vaginal irrigation for the prevention of peripartal infection: A placebo-controlled randomized clinical trial. American Journal of Obstetrics and Gynecology 1997;176:617-22.
156. Mercer BM, Miodovnik M, Thurnau GR, et al. Antibiotic therapy for reduction of infant morbidity after preterm premature rupture of the membranes: A randomized controlled trial. Journal of the American Medical Association 1997;278:989-95.
