# Supplementary material for: Composite adverse outcomes in obstetric studies: a systematic review
Source: BMC Pregnancy Childbirth. 2021 Feb 5;21:107. doi: 10.1186/s12884-021-03588-w (PMC7863533; doi:10.1186/s12884-021-03588-w)

Supplementary Data 3: Components outcomes included in maternal wound-related composite outcomes

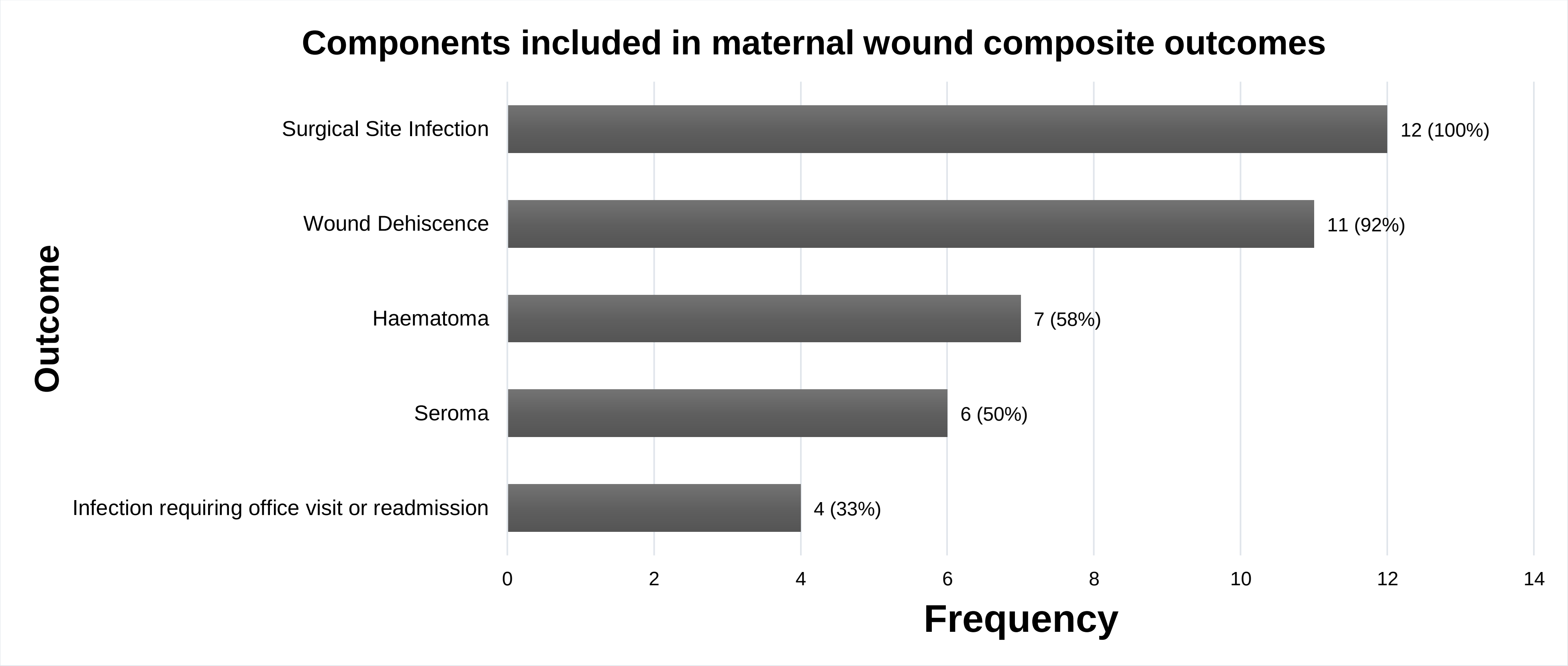

Supplement: Supplementary file 3 — Additional file 3: Supplementary Data 3. Components outcomes included in maternal wound-related composite outcomes. [file 12884_2021_3588_MOESM3_ESM.pdf]
