# Supplementary material for: Composite adverse outcomes in obstetric studies: a systematic review
Source: BMC Pregnancy Childbirth. 2021 Feb 5;21:107. doi: 10.1186/s12884-021-03588-w (PMC7863533; doi:10.1186/s12884-021-03588-w)
Supplement: Supplementary file 4 — Additional file 4: Supplementary Data 4. Condition or organ-specific maternal composite outcomes. [file 12884_2021_3588_MOESM4_ESM.pdf]

Supplementary Data 4: Condition-specific maternal composite outcomes

**Condition-specific morbidity**	**Infectious morbidity** (n=6)	**Bleeding** (n=2)	**Respiratory morbidity** (n=1)	**Perineal trauma** (n=1)	**Labour** (n=1)
**Components (frequency of occurrence)**	• Endometritis (5)<br>• Wound infection (3)<br>• Pneumonia (2)<br>• Death<br>• WHO Stage IV HIV<br>• Urinary Tract Infection<br>• Sepsis<br>• Septic Thrombophlebitis<br>• Pelvic Abscess<br>• Pyelonephritis<br>• Meningitis<br>• Chorioamnionitis<br>• CD4 count > 200	• 4 gm/l drop in haemoglobin<br>• Transfusion of at least 2 units < 48 hours<br>• 500ml of blood loss<br>• Additional use of uterotonics	• Pulmonary oedema<br>• Chest pain<br>• Shortness of breath<br>• Hypotension	• Episiotomy<br>• 2/3/4 lacerations	• Caesarean section<br>• Operative vaginal delivery<br>• Induction of Labour<br>• Augmentation<br>• Pharmacological pain relief

WHO, World Health Organization; * where there are no numbers in parentheses, n=1
